# RUVBL1 and RUVBL2 are druggable MYC effector regulators in neuroblastoma cells

**DOI:** 10.1016/j.isci.2026.115236

**Published:** 2026-03-05

**Authors:** Joachim Tetteh Siaw, Arne Claeys, Wei-Yun Lai, Marcus Borenäs, Elien Hilgert, Sarah-Lee Bekaert, Ellen Sanders, Irem Kaya, Jo Van Dorpe, Frank Speleman, Kaat Durinck, Bengt Hallberg, Ruth H. Palmer, Jimmy Van den Eynden

**Affiliations:** 1 Department of Human Structure and Repair, Anatomy and Embryology Unit, Ghent University, Ghent, Belgium; 2 Cancer Research Institute Ghent, Ghent, Belgium; 3 Department of Medical Biochemistry and Cell Biology, Institute of Biomedicine, Sahlgrenska Academy, University of Gothenburg, Gothenburg, Sweden; 4 Department of Biomolecular Medicine, Ghent University, Ghent, Belgium; 5 Department of Diagnostic Sciences, Ghent University, Ghent, Belgium

**Keywords:** biological sciences, molecular biology, neuroscience, molecular neuroscience

## Abstract

High-risk neuroblastoma is characterized by *MYCN* amplification and high *MYCN* or *MYC* gene expression. These patients have a poor prognosis and there is an urgent need for more effective drugs. While strategies to develop inhibitors that directly target the MYC proteins have remained largely unsuccessful, recent preclinical studies have identified ATR, a key protein of the DNA damage response, as a promising alternative therapeutic target. Here, we identified a strong RUVBL1 and RUVBL2 signature in transcriptomics data derived from different *MYCN*-driven mice tumors treated with ATR inhibitors. The RUVBL proteins form a complex with ATPase activity that has broad cellular functions and we demonstrate that pharmacological inhibition of this protein complex results in a strong reduction of MYC(N) signaling, cell-cycle arrest, DNA damage, and apoptosis. We confirmed the association with *MYCN* and identified the *RUVBL* genes as independent prognostic biomarkers in human primary neuroblastoma data.

## Highlights

• The RUVBL ATPase regulates MYC(N) effector functions in neuroblastoma cells
• Pharmacological inhibition results in DNA damage, cell-cycle arrest, and apoptosis
• RUVBL1 and RUVBL2 expression are independent predictors of neuroblastoma prognosis

## Graphical abstract

**Figure:**
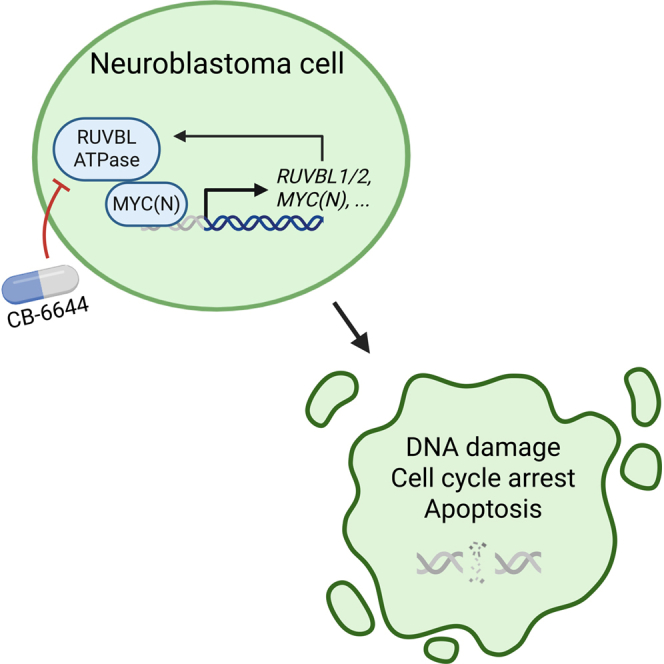

## Introduction

Neuroblastoma (NB) is the most common cancer in infancy. It arises from neural crest derived immature sympathoblasts and occurs along the sympathetic chain ganglia and in the adrenal gland.^1^^,^^2^ Despite the improvement in survival over the years, more than 50% of high-risk patients relapse despite intensive multimodal therapies.^3^^,^^4^^,^^5^ Additionally, severe life-threatening toxicities occur in many high-risk patients.^5^^,^^6^ Hence, safer and more effective drugs are urgently needed.

NB cells are genomically characterized by recurrent patterns of distinct segmental chromosomal aberrations including deletions in 1p and 11q, gains in 2p and 17q, and *MYCN* amplification.^7^^,^^8^^,^^9^^,^^10^^,^^11^ *MYCN* amplification and high *MYC* expression are associated with the majority of high-risk NB cases.^12^^,^^13^^,^^14^^,^^15^ While pharmacological inhibition of MYCN (n-Myc) and MYC (c-Myc) would be a highly effective therapeutic strategy, the development of such inhibitors has proven challenging, due to the lack of a defined binding pocket.^16^^,^^17^^,^^18^

Previous research identified the DNA damage response (DDR) factors ATR and CHK1 as promising therapeutic targets in high-risk NB.^19^^,^^20^^,^^21^^,^^22^ Here, we identified RUVBL1 (Pontin) and RUVBL2 (Reptin) as key mediators in the therapeutic response to ATR inhibition. Both RUVBL1 and RUVBL2 (commonly referred to further as RUVBL1/2) are members of the AAA+ (ATPase associated with diverse cellular activities)-family of ATPases and form heterohexameric or heterododecameric protein complexes that are involved in a broad range of cellular activities, including transcriptional co-activation, epigenetic regulation of gene expression, cell proliferation, DNA repair, regulation of telomerase activity, and senescence.^23^^,^^24^^,^^25^^,^^26^^,^^27^^,^^28^^,^^29^ Although the RUVBL protein complex participates in gene-regulatory mechanisms, it lacks sequence-specific DNA-binding domains and therefore exerts its functions primarily by acting as a cofactor for various transcription factors or DNA-binding proteins, including MYC, RNA polymerase II, E2F, and β-catenin.^23^ We experimentally demonstrate that pharmacological inhibition of RUVBL1/2 using CB-6644 results in reduced MYC and MYCN activity, DNA damage and apoptosis in several NB cell lines and clinically validated these findings in large NB patient datasets by showing that *RUVBL1/2* are strong predictors of NB prognosis, independent of *bonafide* biomarkers.

## Results

### Identification of the RUVBL1/2 complex as a putative therapeutic target in neuroblastoma

**Figure 1:**
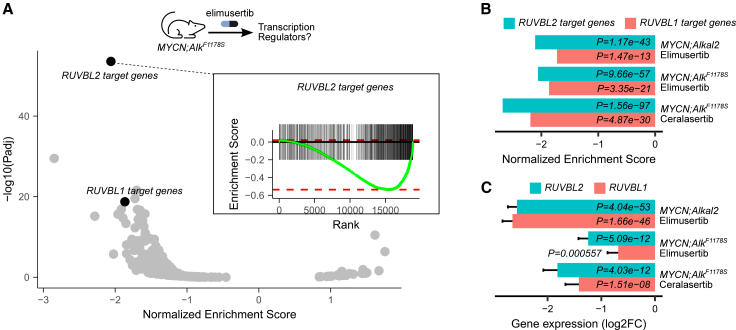
Transcriptional regulator gene set enrichment analysis on NB mice treated with ATR inhibitors Differential gene expression (DGE) data obtained from *Th-MYCN;Alk*^*F1178S*^ (*MYCN;Alk*^*F1178S*^) and *Th-MYCN;Rosa26_Alkal2* (*MYCN;Alkal2*) driven NB mice tumors treated with the ATR inhibitors elimusertib and ceralasertib (both 25 mg/kg per day and after 3 days of treatment) were obtained from previous studies.^19^^,^^21^ (A) Volcano plot showing GSEA results performed using transcriptional regulator target genes on elimusertib treated *Th-MYCN;Alk*^*F1178S*^ tumors as indicated. Running score plot for RUVBL2 target genes shown on inset. (B) Bar plot showing normalized enrichment scores (NES) and corresponding *p* values for RUVBL1 and RUVB2 target genes in 3 experimental conditions as indicated. *p* values calculated using a permutation test as implemented in the fgsea package. (C) Bar plot showing differential *RUVBL1* and *RUVBL2* gene expression results (log2 fold change and *p* values) in 3 experimental conditions as indicated. *p* values calculated using the Wald Statistic, as implemented in the DESeq2 package. Error bars represents the standard error.

We previously demonstrated that therapeutic inhibition of ATR in genetically engineered *ALK*/*MYCN*-driven NB mice models is a highly effective treatment strategy.^19^^,^^21^ To identify other putative treatment targets for NB, we mined our published ATR inhibitor-treated mice tumor RNA-seq data for key transcriptional regulators involved in the observed treatment effects. A gene set enrichment analysis (GSEA) was performed using previously published transcriptional regulator targets that were identified by Chip-seq on mouse embryonic stem cells.^30^ The target genes of the transcriptional co-factor RUVBL2 were the most significantly depleted gene set in *Tg(Th-MYCN)/0; Alk*^*F1*^^*1*^^*78F*^*/+* (hereafter referred to as *Th-MYCN;Alk*^*F1178S*^) mice treated for 3 days with the ATR inhibitors elimusertib (25 mg/kg; NES = −2.1, *p* = 9.66e-57) or ceralasertib (25 mg/kg; NES = −2.7, *p = 1.56e-97*), as well as in elimusertib-treated *Tg(Th-MYCN)/0; Rosa26_Alkal2/+* (hereafter referred to as *Th-MYCN*;*Alkal2*) mice (NES *=* −2.1, *p = 1.17e-43*; Figures 1A and 1B). RUVBL2 forms a protein complex with RUVBL1 and, as expected, a strong depletion of the RUVBL1 targets was observed (NES = −1.9 or lower, *p* = 1.47e-13 or lower, Figures 1A and 1B). Apart from the downregulation of RUVBL targets, we also observed a significant downregulation of the *RUVBL* genes themselves in all 3 experimental conditions (*p* < 0.001; Figure 1C). These results suggest that the treatment effects observed upon ATR inhibition could be at least partially mediated through RUVBL downregulation, identifying the latter as a putative (combinatorial) drug target for neuroblastoma.

### RUVBL1 and RUVBL2 are druggable dependency genes in neuroblastoma cell lines

**Figure 2:**
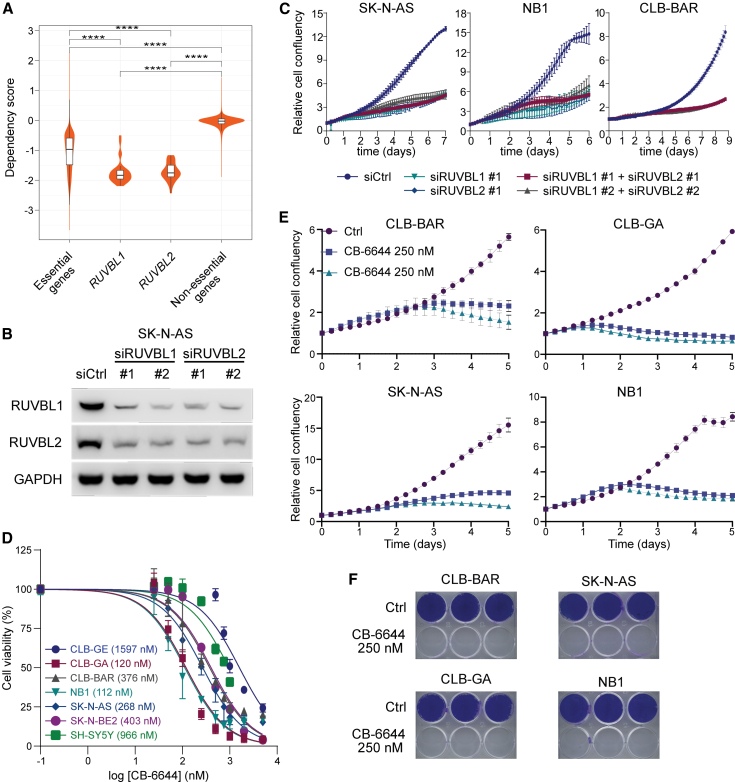
Pharmacological inhibition of RUVBL1/2 ATPase activity in NB cell lines (A) Violin plots comparing DepMap *RUVBL1* and *RUVBL2* dependency scores from 34 NB cell lines to sets of essential and non-essential genes. ∗∗∗∗*p* value <0.0001, two-sided Wilcoxon rank-sum test. (B) Western blot showing transient siRNA-mediated knockdown of *RUVBL1* and *RUVBL2* after 3 days of transfection. Scrambled siRNA was used as control (siCtrl). (C) Time-dependent effect of siRNA-mediated knockdown of *RUVBL1* and/or *RUVBL2* on NB cell line proliferation, as monitored by live scanning for cell confluency at regular time intervals with IncuCyte S3 system. Cell growth was normalized relative to the first scan at time zero. Results are mean ± SEM of 3 independent biological replicates. (D) Cell viability dose dependency curves after treatment with the RUVBL1/2 inhibitor CB-6644 in 7 different NB cell lines, as indicated. Mean IC50 values are indicated for each cell line (2–6 biological replicates). Cell viability was determined by resazurin assay. (E) Time-dependent effect of CB-6644 (250 nM or 500 nM) treatment on NB cell line proliferation, monitored as in C. (F) Long-term (14 days) effect of CB-6644 (250 nM) on NB cell growth. Cells were stained with crystal violet. See Figure S9 for western blot source data.

To find support for the functional roles of *RUVBL1/2* in NB, we first analyzed publicly available gene dependency scores of 34 NB cell lines, with negative scores indicating gene dependency.^31^ Interestingly, both *RUVBL1* (median score = −1.83) *and RUVBL2* (median score = −1.75) had significantly lower scores than a previously identified set of 1910 essential genes (median score = −1.00; *p* < 0.0001; two-sided Wilcoxon rank-sum test; Figure 2A).

To further evaluate the specific cellular effects of RUVBL1/2 downregulation, we carried out transient knockdown using two independent pairs of small interfering RNAs (siRNAs) in the CLB-BAR, NB1, and SK-N-AS NB cell lines. Each siRNA efficiently knocked down its target, *RUVBL1* or *RUVBL2*. Interestingly, knockdown of *RUVBL1* also resulted in downregulation of RUVBL2 and vice versa (Figure 2B). We monitored cell growth for 1 week and observed a decreased cell number after 3–5 days of treatment as compared to control conditions in all studied cell lines (Figure 2C).

Based on these results, we aimed to determine whether RUVBL is a putative therapeutic target in NB and treated seven different NB cell lines, with diverse genetic backgrounds (Table S1), with a recently reported small molecule inhibitor, CB-6644, which specifically blocks the ATPase activity of the RUVBL1/2 complex.^32^ We first determined the sensitivity of the different cells to CB-6644 using a resazurin cell viability assay (Figure 2D) and then assessed the impact on cell confluency upon CB-6644 (250 nM or 500 nM) treatment for the 4 most sensitive cell lines (CLB-BAR, CLB-GA, SK-N-AS, and NB1). This treatment resulted in a complete block of NB cell growth starting 1–3 days after treatment initiation, 1–2 days earlier than after siRNA treatment, depending on the cell line (Figures 2E and 2F).

### Pharmacological RUVBL inhibition is characterized by reduced MYC signaling in NB cells

To better understand the mechanisms underlying the phenotypic response to pharmacological RUVBL inhibition, we performed RNA-seq of *MYCN*-driven CLB-BAR and *MYC*-driven SK-N-AS NB cells after treatment with CB-6644 (250 nM) for 24, 48, and 72 h. The transcriptional response increased progressively from 24 to 72 h and was more pronounced in CLB-BAR (1781 differentially expressed (DE) genes after 72 h) as compared to SK-N-AS cells (989 DE genes; Figures S1A and S1B).

**Figure 3:**
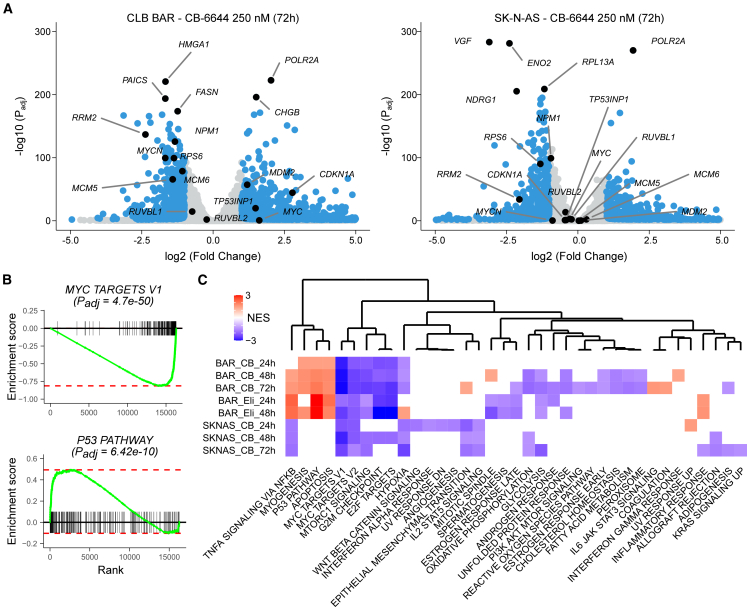
Transcriptomic response to CB-6644 treatment of NB cells CLB-BAR and SK-N-AS NB cells were treated for 72 h with CB-6644 250 nM and differential gene expression (DGE) was determined. (A) Volcano plot for both cell lines as indicated. Differentially expressed genes (threshold log2FoldChange of ±1 at 1% FDR) indicated in blue with top up/downregulated and genes discussed in main text labeled. *p* values calculated using the Wald Statistic, as implemented in the DESeq2 package. (B) GSEA running score plots for 2 Hallmark gene sets in CLB-BAR cells as indicated. *p* values calculated using a permutation test as implemented in the fgsea package. (C) GSEA results with heatmap comparing normalized enrichment values (NES, color key) for both cell lines after treatment with CB-6644 (CB; 250 nM) and elimusertib (Eli; 50 nM) as indicated. Gene sets that were not significant (Padj <0.05) were left blank. Columns (gene sets) were hierarchically clustered as indicated in dendrogram. See Table S2 for detailed DGE and GSEA results.

In CLB-BAR cells 627 genes were downregulated after 72 h of CB-6644 treatment, including *MYCN*, *RRM2*, *RPS6*, *MCM5-*6, and *NPM1* (Figure 3A). A Hallmark GSEA indicated a strong enrichment for MYC target genes (*P*_*adj*_ = 4.7e-50; Figure 3B), MTORC1 signaling and the cell cycle-related targets of E2F transcription factors and G2/M checkpoints, strikingly similar to what we observed previously upon ATR inhibition with elimusertib (Figure 3C).^19^^,^^21^ In SK-N-AS cells (517 genes downregulated after 72 h), similar though overall weaker enrichments were observed as compared to CLB-BAR cells upon CB-6644 treatment (Figure 3C). A reactome pathway GSEA confirmed the enrichment for genes involved in cell cycle control, mainly in CLB-BAR cells (Figure S1C). Additionally, strong enrichments were observed for several RNA-related processes (e.g., RNA metabolism, translation, rRNA processing, and nonsense-mediated decay) in both cell lines (Figure S1C and Table S2).

A different pattern was observed for the upregulated genes (1154 and 472 genes in CLB-BAR and SK-N-AS cells, respectively). While the hallmark P53 (*P*_*adj*_ = 6.4e-10) and apoptosis pathways were strongly enriched in CLB-BAR cells (Figure 3B and 3C), similar to what we observed upon elimusertib treatment, these enrichments were remarkably absent in SK-N-AS cells (*P*_*adj*_ > 0.05; Figure 3C). Notably, *POLR2A* was the most upregulated and fastest responding gene in both cell lines (Figures 3A and S1B).

After siRNA knock down of *RUVBL1* or *RUVBL2* in CLB-BAR cells, a similar downregulation of MYC targets and upregulation of the TP53 pathway was observed, although the overall transcriptional response was rather weak (low logFC values) (Figure S2A).

### Bidirectional transcriptional regulation between MYC(N) and RUVBL1/2 in NB cells

**Figure 4:**
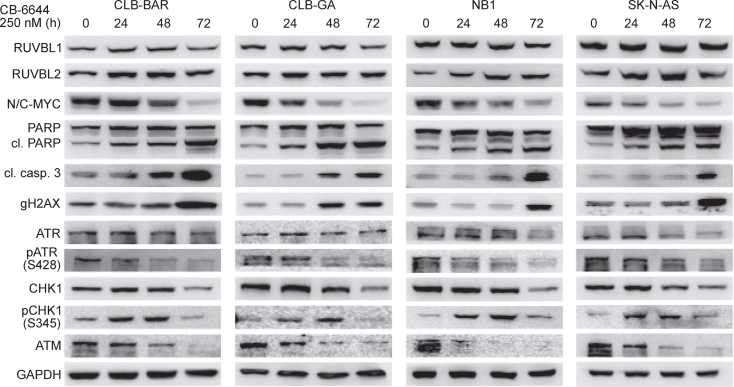
Experimental validation of transcriptomic responses upon CB-6644 treatment of NB cells Western blots showing the effect of different CB-6644 (250 nM) treatment duration on (phospho-) protein expression in 4 NB cell lines as indicated. N/C-MYC denotes MYCN for CLB-BA, CLB-GA, and NB1, and MYC for SK-N-AS and were detected by two independent antibodies. Cl, cleaved. See Figure S10 for western blot source data.

We next aimed to validate this NB cellular response upon CB-6644 treatment. We first confirmed the significant downregulation of *MYCN* (CLB-BAR cells) and *MYC* (SK-N-AS cells) at the transcriptional level using quantitative PCR (Figure S3A). Similar to what we observed in the RNA-seq data, the mRNA reduction was weaker in SK-N-AS (28% lower after 72 h) as compared to CLB-BAR cells (61% lower). Western blotting confirmed this lower MYC(N) expression at the protein level in 4 different NB cell lines (Figure 4). Similar results were obtained after siRNA mediated knockdown of *RUVBL1* or *RUVBL2*, confirming *RUVBL1/2* specificity (Figure S2B).

To determine whether this regulation could be bidirectional (i.e., MYCN regulating *RUVBL1/2* expression), we first performed an RUVBL1/2 western blot after inducing *MYCN* in SHEP MYCN cells. Expression of RUVBL1, and to a lesser extent RUVBL2, was higher 24–48 h after MYCN induction (Figure S3B). We then queried the cell line exploration web application of NB (CLEAN^33^) and retrieved RNA-seq data from 2 other, previously published studies on SHEP-MYCN cells, one after 6 h of MYCN induction (Poon et al.^34^), the other after 16 h (Agarwal et al.^35^). Both studies confirmed increased *RUVBL1* and *RUVBL2* expression after *MYCN* induction, although this was only significant for *RUVBL1* (*p* < 0.01 for both studies) and not for *RUVBL2* (Figure S3C). Interestingly, a GSEA on these data indicated an upregulation of the same cluster of gene sets (MYC targets, MTORC1 signaling, G2M checkpoints, and E2F targets) that we observed to be downregulated upon pharmacological RUVBL inhibition (Figure S3D). Additionally, when analyzing previously published gene expression data of samples taken during different stages of mice NB tumor development, we noticed a developmental increase in *Ruvbl1* and *Ruvbl2* expression in homozygous *Th-MYCN* mice but not in wild-type mice (Figure S3E), supporting a role of RUVBL1/2 in mediating MYCN effects.

**Figure 5:**
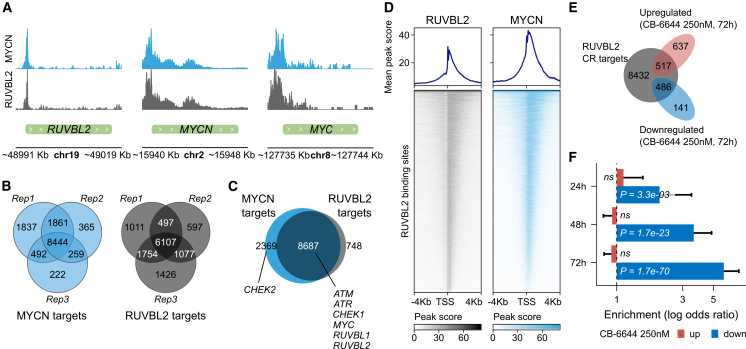
RUVBL2 and MYCN CUT&RUN results in CLB-BAR NB cells (A) MYCN (top, blue) and RUVBL2 (bottom, gray) CUT&RUN peaks at the *RUVBL2*, *MYCN*, and *MYC* gene location in CLB-BAR cells, as indicated. (B) Venn diagram indicating the number of significant MYCN and RUVBL2 CUT&RUN peaks (targets) for 3 replicates. The targets that were identified by minimally 2 out of 3 replicates were used for further analysis (reported in panels C, E, and F). (C) Venn diagram indicating overlap between protein-coding gene targets determined with MYCN and RUVBL2 CUT&RUN. (D) Heatmaps showing peaks scores at the transcription start (TSS) ±4 kb of RUVBL2 and MYCN targets for all RUVBL2 binding sites. (E) Venn diagram showing overlap between the protein-coding RUVBL2 CUT&RUN targets and up- or downregulated genes following 72 h of CB-6644 (250 nM) in CLB-BAR cells, as indicated. (F) Enrichment for RUVBL2 CUT&RUN targets of up- and downregulated genes following different CB-6644 (250 nM) treatment durations as indicated. Odds ratios and corresponding *p* values calculated using Fisher’s exact test. See Table S3 for detailed MYCN and RUVBL2 CUT&RUN results. CUT&RUN performed in triplicate. Results in A and D are from replicate 1. See Figure S4 for the results from replicates 2–3.

To better understand the mutual transcriptional regulatory interplay between MYC(N) and RUVBL, we mapped the genome-wide MYCN and RUVBL binding sites with a CUT&RUN experiment in CLB-BAR cells, using antibodies directed against the MYCN as well as the RUVBL2 protein. This experiment confirmed MYCN binding at the *RUVBL1* and *RUVBL2* promotor and RUVBL2 binding at the *MYC* as well as the *MYCN* promotor, although the latter was only significant in 1 out of 3 replicates (Figures 5A, S4, and Table S3). Strikingly, both MYCN and RUVBL2 had a highly similar transcriptional profile with 8687 out of 9435 (92%) protein-coding RUVBL2 targets that were also targeted by MYCN (Figures 5B–5D and S4). Other targets of both proteins included *ATM*, *ATR*, and *CHEK1*, genes that encode for proteins involved in the DDR (Figure 5C). From the 627 genes that were downregulated after 72 h treatment with 250 nM CB-6644, 486 (78%) were identified as RUVBL2 binding sites by our CUT&RUN experiment (OR = 5.95; *p* = 1.7e-70; Figures 5E and 5F), a pattern that was not observed for the 1154 upregulated genes (OR = 0.91; *p* = 0.14).

### Pharmacological RUVBL inhibition results in DNA damage, S-phase arrest, and apoptosis in NB cells

Next to the downregulated MYC targets, the CB-6644-induced transcriptomic response was further characterized by increased apoptosis signaling and cell cycle alterations (i.e., downregulation of E2F targets and G2M checkpoints), mainly in CLB-BAR cells. This apoptosis induction was confirmed in all examined cell lines by elevated expression of cleaved PARP as well as cleaved caspase 3 (Figure 4). We further confirmed this by increased caspase 3/7 activity as assessed by the caspase-Glo 3/7 apoptosis assay in CLB-GA and SK-N-AS cell lines (Figure S5A). Relatedly, when assessing the NB cell cycle using propidium iodide staining and flow cytometry upon treatment of CLB-GA and SK-N-AS cells with CB-6644, a significant S-phase arrest was observed in both CLB-GA (*p* = 0.013) and SK-N-AS cells (*p* = 0.0072) after 48 h of treatment (Figure S5B).

Given the putative role of RUVBL1/2 in mediating at least part of the therapeutic effects we previously observed upon inhibition of ATR (Figure 1), in combination with the previously suggested PIKK interactions,^36^^,^^37^ we examined whether RUVBL inhibition resulted in an alteration of the DDR. After 48 h–72 h of CB-6644 treatment we observed decreased phosphorylation levels of pATR^S428^ as well as the main ATR target pChk1^S345^ (Figure 4). This reduced ATR-CHK1 signaling coincided with increased expression of the DNA damage marker γH2AX as well as the transcriptomic, proteomic and functional indicators of apoptosis. Notably, this reduced DDR signaling was preceded by increased phosphorylation of pChk1^S345^ 24 h–48 h after treatment and followed by reduced protein expression of ATM and to a lesser extent also ATR and CHK1 after 72 h (Figure 4). Interestingly, the combined treatment of NB cell lines with low doses of CB-6644 (100 nM) and elimusertib (20 nM) resulted in more pronounced inhibition of cell growth, stronger reductions in MYC(N) as well as increased expression of γH2AX, cleavage of caspase 3 and PARP as compared to single drug-treated cells (Figure S6).

### *RUVBL1* and *RUVBL2* are *MYCN*-independent prognostic biomarkers in human neuroblastoma

Our previous results suggest that *RUVBL1* and *RUVBL2* are therapeutically targetable dependency genes in NB. To find clinical evidence for such a role we focused on different sources of primary NB tumors and data.

**Figure 6:**
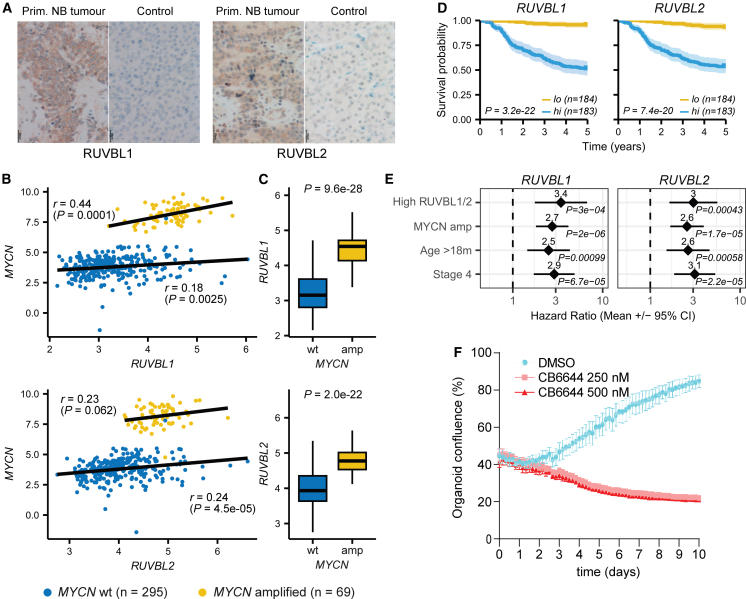
Clinical validation of the prognostic and putative therapeutic relevance of the *RUVBL* genes (A) Immunohistochemical staining for RUVBL1 and RUVBL2 in human NB tumor tissue sections as indicated. Normal pancreatic tissue was used as negative control for antibody specificity. Images are representative of 3 independent NB tumors and 2 independent pancreatic stained tissues. (B–E) Analysis of publicly available primary neuroblastoma data (data from *Cangelosi* et al.^38^; *n* = 364). (B) Correlation plots between *RUVBL1* (top), *RUVBL2* (bottom), and *MYCN* gene expression (log2 normalized counts) for *MYCN* amplified and *MYCN* wild-type tumors. Linear regression line and Pearson’s correlation coefficient indicated. (C) Boxplots comparing *RUVBL1* (top) and *RUVBL2* (bottom) expression between *MYCN* amplified and *MYCN* wild-type tumors. *p* value calculated using two-sided Wilcoxon rank-sum test. (D) Kaplan-Meier survival plots comparing overall survival between patients with high and low *RUVBL1* and *RUVBL2* as indicated. High/low *RUVBL1/2* expression defined based on median gene expression. *p* value calculated using log-rank test. (E) Forest plots comparing hazard ratios ±95% confidence intervals for 4 variables as indicated. Results were obtained using a Cox proportional hazards multivariate regression analysis. (F) Time-dependent effect of CB-6644 (250 nM or 500 nM as indicated) on human NB PDX organoid growth. Organoid growth was monitored by scanning confluency at regular intervals with IncuCyte live cell analysis system. Results are mean ± SD of three technical replicates. Graph is representative of 3 biological repeats with different organoid seeding densities.

We first confirmed protein expression of RUVBL1 and RUVBL2 in primary NB cancer cells using immunohistochemistry (Figure 6A). We then focused on a large set (*n* = 364) of previously published primary NB RNA-seq and associated clinical data. Similar to what we observed in cell lines, *MYCN* and *RUVBL1/2* expression were positively correlated, both in *MYCN* amplified and in *MYCN* wild-type tumors (Pearson’s *r* between 0.18 and 0.44; Figure 6B). As expected, *MYCN* amplified tumors had significantly higher *RUVBL1* (*p* = 9.6e-28) and *RUVBL2* (*p* = 2.0e-22) expression as compared to *MYCN* wild-type tumors (Figure 6C). Additionally, significantly worse survival was observed between tumors with high (above median) expression of *RUVBL1* (*p* = 3.2e-22; 5-year survival 51%) or *RUVBL2* (*p* = 7.4e-20; 5-year survival 54%) as compared to tumors with low expression of these genes (5-year survival 93% or higher; Figure 6D)*.* Remarkably, despite the strong correlation between *RUVBL1/2* expression and *MYCN* amplification state, high *RUVBL1* and *RUVBL2* expression were identified as strong and *MYCN* amplification-independent prognostic biomarkers of NB outcome. Indeed, a Cox multivariate proportional hazards regression that considered higher-than-median *RUVBL1* or *RUVBL2* expression together with *bonafide* prognostic NB biomarkers as covariates (i.e., tumor stage, age at diagnosis, and *MYCN* amplification) indicated significantly elevated hazard ratios for both high *RUVBL1* (HR = 3.4*; p* = 3.0e-04*)* and high *RUVBL2 (*HR = 3.0*; p* = 4.3e-04) expression (Figure 6E)*.* A positive correlation was also observed between expression of the *RUVBL* genes and *ALK* in *MYCN* wild-type, but not in *MYCN* amplified NB patients. The association between high *RUVBL* expression and survival was not affected after adding *ALK* expression state to the cox multivariate model (Figure S7).

Finally, we explored the therapeutic potential of CB-6644 RUVBL inhibition by treating organoids derived from a human high-risk, *MYCN* amplified NB patient. Similar to our cell line results, organoid growth was completely abrogated after treatment with CB-6644 250 nM or 500 nM (Figures 6F and S8). Altogether, these reinforce *RUVBL1/2* as dependency genes in NB pathogenesis.

## Discussion

The DDR is a target of major interest in high-risk NB with several recent studies reporting highly promising results when therapeutically targeting key molecules such as ATR, AURKA, or CHK1 in NB animal models.^19^^,^^20^^,^^21^^,^^22^ In this study, we discovered a strong *RUVBL1* and *RUVBL2* signature in transcriptomic data obtained from tumors treated with ATR inhibitors in *ALK*/*MYCN*-driven NB mice models. *RUVBL1* and *RUVBL2* form a protein complex with ATPase activity that can be inhibited by the small molecule inhibitor CB-6644. We demonstrated that CB-6644 inhibition of RUVBL1/2 activity resulted in reduced MYC(N) signaling, apoptosis, cell-cycle arrest, and increased DNA damage. In primary NB data, we observed a strong association between high *RUVBL1/2* expression and bad outcome, independent of the main risk factors (*MYCN* amplification status, age, and stage). Our results suggest that RUVBL1/2 is a therapeutically targetable dependency gene and important prognostic biomarkers in NB.

One of the most striking signals we observed upon pharmacological RUVBL1/2 inhibition was the downregulation of MYC targets. These targets are regulated by the MYC family of transcription factors, which includes MYCN (n-MYC), one of the most potent oncogenes in high-risk NB. Their lack of apparent surfaces for small molecule binding makes them difficult to target therapeutically,^39^ and our results identify the RUVBL1/2 complex as an indirect therapeutic MYC target in NB, similar to recent findings in pancreatic cancer.^40^ A positive correlation was found between *MYCN* and *RUVBL1/2* mRNA levels in NB cell lines as well as primary NB tumors, suggesting an interdependency at the transcriptional level, in line with previous reports.^23^^,^^41^ Interestingly, we confirmed that this transcriptional regulation was bidirectional and demonstrated MYCN binding at *RUVBL1/2* promotor regions and RUVBL2 binding at the *MYC(N)* promotor. The strong overlap between the broad range of RUVBL and MYCN transcriptional targets is in line with the previously suggested role of RUVBL1/2 as an MYC cofactor that is essential for chromatin association.^40^ The majority (78%) of genes that were downregulated upon pharmacological inhibition with CB-6644 were identified as bindings sites of the RUVBL protein with RUVBL2 CUT&RUN, supporting RUVBL specificity of the inhibitor. However, while the overall transcriptomic response upon siRNA inhibition was similar (i.e., downregulation of MYC targets identified using GSEA), differential expression was rather weak at the individual gene level. This weaker response likely reflects incomplete siRNA knockdown (Figure 2B) as well as the time required for siRNA-mediated protein depletion compared to immediate pharmacological inhibition. The latter was also suggested by the 1–2 days delay in cell growth reduction we observed in siRNA treated cells as compared to CB-6644 treated cells (Figure 2C).

The transcriptomic response upon RUVBL1/2 inhibition was similar to what we observed upon ATR inhibition with a reduction of MYC, E2F, MTORC1, and G2M checkpoint signaling and increased (p53-related) apoptosis signaling.^19^ Whether or not ATR influences RUVBL directly or indirectly (e.g., as a response to increased DNA damage) remains to be determined. Conversely, we identified ATR and ATM as direct transcriptional targets of MYCN and RUVBL2. Together with the previously suggested role of the RUVBL1/2 complex in the stabilization of PIKKs,^42^ these different mechanisms likely explain the reduced MTORC1 signaling and decreased ATR and ATM protein expression we observed after 24–48 h of RUVBL inhibition. While we also observed ATR dephosphorylation after RUVBL inhibition, it remains to be determined whether this is a result of a direct signaling interaction or secondary to decreased ATR protein expression.

One of the major effectors of MTORC1 signaling is the ribosomal protein S6 kinase (S6K). This protein controls protein synthesis through phosphorylation of the ribosomal protein S6 (*RPS6* gene) which putatively explains the reduced rRNA processing, RNA metabolism, and translation activities we observed in our transcriptome data upon RUVBL inhibition. Interestingly, our study indicated a strong and consistent downregulation of *RPS6* and in a previous protein kinase analysis we found strongly reduced kinase activity of several related ribosomal kinases upon ATR inhibition.^21^^,^^33^

Recent evidence suggests that RUVBL1 is involved in MYC multimerization near stalled replication forks, protecting them against RNA polymerase II (Pol II)-mediated replication stress, a hallmark of high-risk-NB.^43^ Relatedly, the RUVBL1/2 complex has been shown essential for both the assembly and the regulation of the Pol II complex.^23^^,^^42^ Strikingly, the Pol II encoding *POLR2A* gene was the earliest (24h) and strongest upregulated gene, which could thus represent a compensatory mechanism resulting from decreased Pol II activity.

A role of RUVBL has been suggested in many cancer types, including colorectal cancer, breast cancer, lung cancer, and many others.^36^^,^^40^^,^^42^^,^^44^^,^^45^ In general, these studies found tumor-promoting effects of *RUVBL1* and/or *RUVBL2* and a link between high RUVBL1/2 expression and poor clinical outcome. Our study demonstrates that RUVBL1 and RUVBL2 are also clinically relevant and targetable dependency genes in NB. Only 2 studies hinted toward a role of RUVBL in NB previously: *Zhan* et al. reported on the involvement of RUVBL2 in mediating SK-N-DZ NB cell death upon histone deacetylase inhibition,^46^ while very recently, *RUVBL1* was found to be part of a multigene prognostic signature in NB.^47^ Interestingly, our results suggest that the apoptosis induction upon RUVBL inhibition is mediated by p53 and non-p53 pathways. Indeed, while we only found a transcriptomic signal of p53-induced apoptosis in CLB-BAR cells and not SK-N-AS cells (*TP53* is deleted in these cells^48^), all cell types examined responded with a clear induction of apoptosis and decreased cell growth upon experimental RUVBL inhibition.

RUVBL proteins are essential genes and future *in vivo* evaluations of pharmacological inhibition raise toxicity concerns. Careful evaluation of the therapeutic window and rational treatment combinations (e.g., with ATR inhibition) will therefore be required to balance efficacy with tolerability. Encouragingly, prior *in vivo* investigations with CB-6644 have reported limited toxicity, suggesting that selective inhibition strategies may be feasible under specific conditions.^32^^,^^40^

In conclusion, our study has identified *RUVBL1* and *RUVBL2* as clinically relevant and therapeutically targetable dependency genes with independent prognostic value in NB. Our results suggest critical interactions between *RUVBL1/2*, *MYC(N)*, and *ATR* and highly converging downstream signaling pathways in NB. While this opens several options toward novel combinatorial treatment strategies, one of the key future challenges will be to identify the most efficacious and least toxic therapeutic strategies in preclinical and later clinical studies.

### Limitations of the study

Our study is limited by the lack of pharmacological RUVBL inhibition in experimental neuroblastoma model organisms. Additionally, while highly converging and putatively synergistic downstream pathways were demonstrated after ATR and RUVBL inhibition, the precise mechanistic link remains to be determined.

## Resource availability

### Lead contact

Requests for further information and resources should be directed to and will be fulfilled by the lead contact, Jimmy Van den Eynden (jimmy.vandeneynden@ugent.be).

### Materials availability

This study did not generate new unique reagents.

### Data and code availability

• RNA-seq and CUT&RUN data have been deposited at ArrayExpress and Gene Expression Omnibus (GEO) and are publicly available as of the date of publication. Accession numbers are listed in the key resources table. Quantified gene expression data are interactively available in the CLEAN web application at https://ccgg.ugent.be/shiny/clean/siaw_2026/.^33^
• Source code used for RNA-seq and public data analyses is available at GitHub https://github.com/CCGGlab/RUVBL. Source code used for CUT&RUN data processing is available at https://github.com/PPOLLabGhent/nf_Pipeline_CUTandRUN.
• Any additional information required to reanalyze the data reported in this paper is available from the lead contact upon request.

## STAR★Methods

### Key resources table

**Table:** 

REAGENT or RESOURCE	SOURCE	IDENTIFIER
**Antibodies**		

Anti-ATM (host rabbit)	Cell Signaling Technology	RRID:AB_2062659
Anti-ATR (host rabbit)	Cell Signaling Technology	RRID:AB_2798347
Anti-Chk1 (host rabbit)	Cell Signaling Technology	RRID:AB_10693648
Anti-Cleaved Caspase-3 (host rabbit)	Cell Signaling Technology	RRID:AB_2341188
Anti-*c*-Myc (host rabbit)	Cell Signaling Technology	RRID:AB_2895543
Anti-GAPDH (host rabbit)	Cell Signaling Technology	RRID:AB_10622025
Anti-IgG (host rabbit)	Cell Signaling Technology	RRID:AB_1550038
Anti-IgG (host goat)	Thermo Fisher Scientific	RRID:AB_1965959
Anti-N-Myc (host rabbit)	Cell Signaling Technology	RRID:AB_10692664
Anti-N-Myc (host mouse)	Santa Cruz	RRID:AB_831602
Anti-PARP (host rabbit)	Cell Signaling Technology	RRID:AB_2160739
Anti-pATR (Ser428; host rabbit)	Cell Signaling Technology	RRID:AB_2290281
Anti-pChk1 (Ser345; host rabbit)	Cell Signaling Technology	RRID:AB_331212
Anti-RUVBL1 (host rabbit)	Sigma-Aldrich	RRID:AB_1856514
Anti-RUVBL1 (host rabbit)	Sigma-Aldrich	RRID:AB_1856516
Anti-RUVBL1 (host rabbit)	Cell Signaling Technology	RRID:AB_2799859
Anti-RUVBL2 (host rabbit)	Novus Biologicals	RRID:AB_3342625
Anti-RUVBL2 (host rabbit)	Sigma-Aldrich	RRID:AB_2685933
Anti-RUVBL2 (host rabbit)	Thermo Fisher Scientific	RRID:AB_2547345
Anti-RUVBL2 (host rabbit)	Cell Signaling Technology	RRID:AB_2797987
Anti-β-Actin (host rabbit)	Cell Signaling Technology	RRID:AB_2223172

**Chemicals, peptides, and recombinant proteins**		

CB-6644	MedChemExpress	Cat# HY-114429
Elimusertib	Selleck Chemicals	Cat# S9864
lipofectamine RNAiMAX	ThermoFisher Scientific	Cat# 13778150
StemPro Accutase	Gibco	Cat# A11105-01
Caspase-Glo® 3/7 Assay kit	Promega	Cat# G8090

**Critical commercial assays**		

Cutana pA/G-MNase	Epicypher	Cat# 15-1016
NEBNext Ultra II	Illumina	Cat# E7645
iScript cDNA synthesis kit	Biorad	Cat# 1708890

**Deposited data**		

RNA-Seq data: pharmacological inhibition	ArrayExpress	E-MTAB-13137
RNA-Seq data: siRNA inhibition	ArrayExpress	E-MTAB-16403
CUT&RUN data	Gene expression omnibus (GEO)	GSE312769
Code to process CUT&RUN data	GitHub	https://github.com/PPOLLabGhent/nf_Pipeline_CUTandRUN
Code to analyze all other data	GitHub	https://github.com/CCGGlab/RUVBL

**Experimental models: Cell lines**		

CLB-BAR	Center Leon Berard, France	–
CLB-GE	Center Leon Berard, France	–
CLB-GA	Center Leon Berard, France	–
SK-N-AS	European Collection of Authenticated Cell Cultures (ECACC)	Cat# 94092302
SH-SY5Y	European Collection of Authenticated Cell Cultures (ECACC)	Cat# 94030304
SK-N-BE(2)	European Collection of Authenticated Cell Cultures (ECACC)	Cat# 95011815
NB1	Japanese Cancer Research Resources Bank (JCRB)	Cat# JCRB0621
SHEP-MYCN	–	–

**Oligonucleotides**		

siRUVBL1, #1: CGAGUGAUGAUAAUCCGGAtt	ThermoFisher Scientific	ID# S16369
siRUVBL1, #2: GAAGUUUACUCAACUGAGAtt	ThermoFisher Scientific	ID# S16370
siRUVBL2, # 1: GGAGAUCCGUGAUGUAACAtt	ThermoFisher Scientific	ID# S21307
siRUVBL2, # 2: GAAACGCAAGGGUACAGAAtt	ThermoFisher Scientific	ID# S21309
Scrambled siRNA	Qiagen	Cat# S103650325
Primer: MYCN: FW: 5′-CTGAGCGATTCAGATGATGAAG-3′	Eurofins Genomics	–
Primer: MYCN: RV: 5′-CCACAGTGACCACGTCGATT-3′	Eurofins Genomics	–
Primer: MYC: FW: 5′-CAGCTGCTTAGACGCTGGAT-3′	Eurofins Genomics	–
Primer: MYC: RV: 5′-AGCTAACGTTGAGGGGCATC-3′	Eurofins Genomics	–
Primer: ACTB: FW: 5′-ATGACCCAGATCATGTTTGAGAC-3′	Eurofins Genomics	–
Primer: ACTB: RV: 5′-CCAGAGGCGTACAGGGATAG-3′	Eurofins Genomics	–

**Software and algorithms**		

Bowtie2 v.2.3.1	Langmead et al., 2012	https://bowtie-bio.sourceforge.net/bowtie2/index.shtml
Deeptools v.3.5.6	Anaconda	https://anaconda.org/channels/bioconda/packages/deeptools/overview
DESeq2 v.1.34.0	Anaconda	https://anaconda.org/channels/bioconda/packages/bioconductor-deseq2/overview
FACSDIVATM S	FlowJoTM v10.8	BD Life Sciences
fGSEA	Anaconda	https://anaconda.org/channels/bioconda/packages/bioconductor-fgsea/overview
FlowJoTM v10.8	BD Life Sciences	https://www.flowjo.com/
GloMax® system	Promega	https://be.promega.com/resources/software-firmware/glomax-systems/glomax-discover-system-software/
HISAT2 v2.1.0	Anaconda	https://anaconda.org/channels/bioconda/packages/hisat2/overview
HTSeq v0.11.2	Anaconda	https://anaconda.org/channels/bioconda/packages/htseq/overview
IncuCyte® S3 Live Cell Analysis system	Sartorius	https://www.sartorius.com/en/products/live-cell-imaging-analysis/live-cell-analysis-instruments/s3-live-cell-analysis-instrument
Limma v.3.50.3	Anaconda	https://anaconda.org/channels/bioconda/packages/bioconductor-limma/overview
MACS2 v2.1.0	Python Package Index	https://pypi.org/project/MACS2/
Picard v4.0.11	Broad Institute	https://broadinstitute.github.io/picard/
Plotgardener v.1.12.0	Anaconda	https://anaconda.org/channels/bioconda/packages/bioconductor-plotgardener/overview
R statistical package v.4.3.1	Anaconda	https://anaconda.org/channels/r/packages/r-base/overview
Survival v.3.4.0	Anaconda	https://anaconda.org/channels/r/packages/r-survival/overview
Survminer v.0.4.9.	Anaconda	https://anaconda.org/channels/r/packages/r-survminer/overview
Trimgalore v.0.6.5	Github	https://github.com/FelixKrueger/TrimGalore

### Experimental model and study participant details

#### Cell culture

Eight different NB cell lines with different genomic alterations were used in this study (Table S1). CLB-BAR, CLB-GE and CLB-GA cells were obtained from The Center Leon Berard, France under MTA. SK-N-AS, SH-SY5Y and SK-N-BE(2) cells were purchased from ATCC. NB1 cells were purchased from JCRB (Japanese Cancer Research Resources Bank). SHEP-MYCN cell lines were kindly provided by Tanmoy Mondal (University of Gothenburg, Sweden). All cell lines were tested for mycoplasma. Cell lines were cultured in complete media, RPMI 1640 supplemented with 10% foetal bovine serum (FBS) and a mixture of 1% penicillin/streptomycin at 37 °C and 5% CO_2_.

#### Human neuroblastoma data

RNA-seq and associated clinical follow-up data were obtained from the previous study of *Cangelosi* et al*.*^38^ All available patients with matched RNA-seq, *MYCN* status and clinical follow-up data were included (*n* = 364), without further considering differences in sex and/or gender in the analyses.

### Method details

#### Cell proliferation and cell viability assays

Cell confluency/proliferation was monitored live using the IncuCyte S3 Live Cell Analysis system (Essen BioScience) for 5 days (after treatment). Rate of cell growth under all conditions were determined using the IncuCyte S3 software.

For CB-6644 IC_50_ determination, cells were seeded in 96-well plates at densities between 3000 and 5000 cells/well, and then treated with increasing concentrations of CB-6644. Cell viability was assessed after 3 days using resazurin assay.

#### Foci formation assay

Cells (1.0 × 10^5^) were seeded in 6-well plates and cultured overnight prior to treatment with 250 nM CB-6644 for 14 days. Cells were washed in PBS and fixed with methanol, followed by staining with 0.2% crystal violet, and washed. Plates were then scanned using Toshiba Studio 2505AC.

#### Apoptosis assay

Cells (7000 per well) were seeded in 96-well plate and cultured overnight. They were then treated with 250 nM CB-6644 for either 48 h or 72 h in three technical and three biological replicates. DMSO was used as negative control. Apoptosis was evaluated by measuring caspase 3 and 7 activities in the treated cells, using the Caspase-Glo 3/7 Assay kit (Promega), following the manufacture’s protocol. Briefly, Caspase-Glo reagents were added to the cells in 96-well plate, incubated for 45 min with gentle shaking. Luminescence was recorded with the GloMax system (Promega).

#### Immunoblotting analyses

To evaluate the effect of CB-6644 or *MYCN* induction on RUVBL1/2 and downstream protein expression, NB cell lines (CLB-BAR, CLB-GA, NB1, SK-N-AS and/or SHEP-MYCN) were treated with 250 nM CB-6644 for 24h, 48h and 72h. Protein lysates were collected by lysing cells in RIPA lysis buffer and protein concentration was measured by BCA assay. Samples were subjected to western blot analyses. Chemiluminescence detection was done using Odyssey Fc Imager (LI-COR).

#### Cell cycle analysis

SK-N-AS and CLB-GA (0.65–1.0 x 10^6^ cells) were seeded in a T-25 flask. After overnight culturing, cells were treated for 48h with CB-6644 at IC50 concentrations (SK-N-AS: 250 nM; CLB-GA: 120 nM) or DMSO control. Cells were washed once with ice-cold PBS before collection in Phosphate-buffered saline (PBS). During each wash step, cells were centrifuged for 5 min at 1200 rpm and supernatant was removed. Cells were washed once in ice-cold PBS before fixation in 70% ethanol for at least 1 h on ice, then washed once with ice-cold PBS and incubated in PBS with ribonuclease A (RNase A, 250 μg/mL) for 1 h at 37°C. Propidium iodide (40 μg/mL) was added, and analysis was performed on a BD LSR II flow cytometer using BD FACSDIVATM Software. The flow cytometry results were analyzed using FlowJoTM v10.8 Software (BD Life Sciences).

#### siRNA-mediated knockdown of RUVBL1 and RUVBL2

NB cells were transfected with Silencer select siRNAs (Life Technologies) against *RUVBL1* [ID #S16369; CGAGUGAUGAUAAUCCGGAtt (siRUVBL1 #1) and S16370; GAAGUUUACUCAACUGAGAtt (siRUVBL1 #2)] and RUVBL2 [ID #S21307; GGAGAUCCGUGAUGUAACAtt (siRUVBL2 #1) and S21309; GAAACGCAAGGGUACAGAAtt (siRUVBL2 #2)] using lipofectamine RNAiMAX transfection reagent (# 13778150, ThermoFisher Scientific). Scrambled siRNA (#S103650325, Qiagen) was used as negative control. Cells were harvested after overnight transfection and seeded (2500–4000) in 96-well plates. The remaining cells were seeded in 6-well plates and cultured for 5 days and lysed in RIPA buffer for western blot analysis. The 96-well plates were monitored for cell proliferation using the IncuCyte S3.

#### Organoid growth assay

Human high-risk NB PDX (NEC005) tumoroid, with *MYCN*-amplification, Chr 2p and 17q gains, and Chr 1p and 3p deletions, were dissociated into single cells using StemPro Accutase (# A11105-01; Gibco). Cells were then seeded at 3 different densities, 3000, 5000 and 7000 cells per well of 384 well plate. After overnight culture, cells were treated with either 250 nM or 500 nM CB-6644 in triplicates. DMSO was used as negative control. Organoid growth was monitored live with IncuCyte S3.

#### MYCN and RUVBL2 CUT&RUN analysis

CUT&RUN coupled with high-throughput DNA sequencing was performed on isolated nuclei using Cutana pA/G-MNase (Epicypher, 15–1016) according to the manufacturer’s manual. Briefly, nuclei were isolated from 0.5 x 10^6^ cells/sample in 100 μL nuclear extraction buffer per sample and incubated with activated Concanavalin A beads for 10 min at 4°C while rotating. Nuclei were resuspended in 50 μL antibody buffer containing a 1:100 dilution of MYCN antibody (RRID:AB_831602), RUVBL2 antibody (RRID:AB_3342625) or control IgG (RRID:AB_1550038), and kept in an elevated angle on a nutator at 4°C overnight. Next, targeted digestion and release was performed with 2.5 μL Cutana pA/GMNase (15–1116) and 100 mM CaCl_2_ for 2 h at 4°C on the nutator. After chromatin release by incubation on 37°C for 10 min, DNA was purified using the CUT&DNA purification kit (14–0050) and eluted in 12 μL of elution buffer. Sequencing libraries were prepared with the NEBNext Ultra II kit (Illumina, E7645), followed by paired-end sequencing on a Nextseq2000 using the NextSeq 2000 P2 Reagents 100 Cycles v3 (Illumina, 20046811). Prior to mapping to the human reference genome (GRCh38/hg38) with bowtie2 (v.2.3.1), quality of the raw sequencing data of CUT&RUN was evaluated using FastQC and adapter trimming was done using TrimGalore (v0.6.5). Quality of aligned reads were filtered using a minimum mapping quality (MAPQ) of 30 and reads with known low sequencing confidence were removed using Encode Blacklist regions. For sample with a percentage duplicated reads higher than 10%, deduplication was performed using MarkDuplicates (Picard, v4.0.11). Peak calling was performed using MACS2 (v2.1.0) taking a q value of 0.05 as threshold and default parameters. The R package *PlotGardener* (v1.12.0) was used for peak tracks visualization. Peak heatmaps were generated using the *computeMatrix* (reference-point set to TSS, -beforeRegionStartLength and -afterRegionStartLengthset to 4 Kb) and *plotHeatmap* function from *deeptools* (v.3.5.6). All CUT&RUN experiments were performed in triplicate.

#### Immunohistochemical staining of human NB samples

Human NB (*n* = 3), and pancreatic tissues (*n* = 2, control) were fixed in 10% neutral buffered formaldehyde and embedded in paraffin. Stainings for RUVBL1 and RUVBL2 were performed on 3-μm-thick sections via an automatic immunostainer (BenchMark Ultra, Ventana Medical Systems). Two anti-RUVBL1 (RRID:AB_1856514, 1:100; RRID:AB_1856516, 1:500) and 2 anti-RUVBL2 (RRID:AB_2685933, 1/500; RRID:AB_2547345, 1:50) were incubated for 32 min. Visualization was achieved with the Ultraview Universal DAB Detection Kit (Ventana Medical Systems). Heat-induced epitope retrieval was performed with Cell Conditioning 1 (Ventana Medical Systems), 64 min at 95°C, except for anti-RUVBL1 antibody RRID:AB_1856516 for which an incubation time of 36 min was used.

#### Data download and processing

Human NB RNA-seq (batch corrected log normalized counts) and related clinical data were obtained from the study of *Cangelosi* et al.^38^ (available at https://www.mdpi.com/2072-6694/12/9/2343/s1).

RNA-Seq differential gene expression data from *Th-MYCN*-driven NB mice models treated with the ATR inhibitors elimusertib or ceralasertib (both 25 mg/kg) were obtained from our previous studies^19^^,^^21^ (available at https://static-content.springer.com/esm/art%3A10.1038%2Fs41467-021-27057-2/MediaObjects/41467_2021_27057_MOESM3_ESM.xlsx and https://www.pnas.org/doi/suppl/10.1073/pnas.2315242121/suppl_file/pnas.2315242121.sd02.xlsx). RNA-Seq data from MYCN-induced SHEP cells and elimusertib-treated CLB-BAR cells were retrieved using CLEAN (https://ccgg.ugent.be/shiny/clean/).^33^

Gene expression profiling microarray data from *MYCN*-driven NB tumor development in the *TH-MYCN* mouse model were obtained from ArrayExpress (accession nr. E-MTAB-3247).^49^ Background correction and quantile normalization of these data were performed using the *Limma* v.3.50.3 *R* package.^50^ Gene expression of *Ruvbl1*, and *Ruvbl2* was evaluated at 1, 2, and 6 weeks. Genes differentially expressed between *Th-MYCN*^+/+^ and wild-type mice at week 6 were determined using *Limma* moderated t-statistics to determine significance in gene expression changes.

Gene effect scores (dependency scores) of 34 NB cell lines, derived from CRISPR knockout screens and published by Broad’s Achilles and Sanger’s SCORE projects, were downloaded from DepMap^31^ (https://depmap.org/portal/download/all/). Negative gene effect scores imply cell growth inhibition and/or death following gene knockout. Non-essential genes have a normalized median score of 0 and predefined common essential genes have a median score of −1. Essential genes (*n* = 1910) were derived from DepMap.

#### Gene set enrichment analysis (GSEA)

Preranked GSEA was performed using the R *fgsea* package (*fgseaMultilevel* function, default parameters) with ranking based on the DEseq2 statistic. Mouse (GTRD transcription factor targets) and human (Hallmark, Reactome) gene sets were downloaded from the Molecular Signatures Database v2023.1.

#### RNA-sequencing and quantitative PCR

CLB-BAR and SK-N-AS NB cell lines were treated with 250 nM CB-6644 for 24, 48 and 72 h. DMSO was used as negative control. RNA was extracted from the cell pellets using the ReliaPrep RNA Miniprep Systems (Promega) and the manufacturers protocol was followed. RNA samples were either used for RNA-seq or quantitative PCR.

RNA sequencing was performed by Biomarker Technologies (BMK, Germany). RNA-Seq paired-end reads (read length 150 base pairs) were aligned to the GRCh38 reference genome using *HISAT2 (v2.1.0)*.^51^ The average alignment efficiency for all samples was 93.4%. Genes were annotated using GENCODE 29 and quantified using *HTSeq (v0.11.2)*.^52^ Further analysis was performed using only coding genes. Differential gene expression was determined using *DESeq2 (v1.34.0)*.^53^ Only expressed genes, defined as genes with a basemean value higher than 10, were considered for further analysis. Genes were considered differentially expressed if their absolute log2 fold change values were above 1 at FDR-adjusted *p* values (*Padj*) below 0.05.

cDNA was synthesized using the iScript cDNA synthesis kit (Biorad) and quantitative PCR was performed on StepOnePlus Real-Time PCR Systems using Power SYBR Green master mix and following primers: MYCN: 5′-CTGAGCGATTCAGATGATGAAG-3′ and 5′-CCACAGTGACCACGTCGATT-3’; MYC: 5′-CAGCTGCTTAGACGCTGGAT-3′ and 5′-AGCTAACGTTGAGGGGCATC-3’; ACTB: 5′-ATGACCCAGATCATGTTTGAGAC-3′ and 5′-CCAGAGGCGTACAGGGATAG-3’.

#### Survival analysis

Overall survival (OS) analyses were performed using the R packages *Survival* (v.3.4.0) and *Survminer* (v.0.4.9). OS curves were plotted by the Kaplan-Meier method. Tumor samples were stratified (high vs. low gene expression) based on median gene (*RUVBL1*, *RUVBL2* or *ALK*) expression. Log rank tests were performed to assess statistical significance.

The multivariate Cox proportional hazards regression model was used to evaluate the prognostic values of genes. Tumor stage (stage 4 vs. stages 1,2,3), age at diagnosis (>18 months vs. < 18 months) and *MYCN* amplification were used as covariates in the multivariate analysis.

### Quantification and statistical analysis

Statistical analyses were performed with R statistical package (v.1.3.1 with the exception of the *plotgardener* package, which was ran on R v.4.4.3). Statistical tests are indicated in the respective sections and figure captions. Multiple testing corrections were performed using the Benjamini-Hochberg method.^54^ The scripts used to produce the results reported in this paper are available at https://github.com/CCGGlab/RUVBL.

## Acknowledgments

This project was funded by grants from the 10.13039/501100006313Swedish Childhood Cancer Foundation (TJ2021-0068 - J.T.S.; PR2022-0029 - R.H.P.; PR2024-0061 - B.H.), the 10.13039/501100004359Swedish Research Council (2023-02433 - R.H.P.; 2021-01192 - B.H.), the 10.13039/501100005009Assar Gabrielssons Foundation (FB22-24 - J.T.S.), 10.13039/100000884Cancer Research Institute, Ghent (YIPOC-2023 - J.T.S.), the Ghent University Special Research Fund (BOF.STG.2019.0073.01 - J.V.d.E.; BOF.GOA.2022.0003.03 - F.S.), 10.13039/501100011851Kom op tegen Kanker (Stand up to Cancer), the Flemish Cancer Society (STI.VLK.2022.0013.01 - A.C.), the 10.13039/501100002794Swedish Cancer Society (CAN24/3527 - R.H.P.; CAN24/3553 - B.H.), 10.13039/501100022721Villa Joep grant (F.S.) and the 10.13039/501100003130Research Foundation Flanders (10.13039/501100003130FWO; FWO.OPR.2023.0063.01 - F.S., J.V.d.E., R.H.P., and V424522N - J.V.d.E.).

## Author contributions

This study was conceptualized and designed by J.T.S. and J.V.d.E. qPCR analysis was performed by W.-Y.L. and M.B. Cell cycle, organoid, and CUT&RUN experiments were conducted by E.H. and E.S. and supervised by F.S. and K.D. Primary NB tumor immunohistochemical staining was performed by I.K. and J.V.D. J.T.S. conducted all other wet-lab experimental analyses. J.T.S., A.C., S.B., and J.V.d.E. performed the data analyses. J.T.S., R.H.P., and J.V.d.E. drafted the manuscript, with subsequent review and editing from all authors.

## Declaration of interests

The authors declare no competing interests.

## Supplemental information

Document S1. Figures S1–S13 and Table S1

Table S2. Differential gene expression and gene set enrichment results, related to Figure 3

Table S3. CUT and RUN results, related to Figure 5
