# Supplementary material for: RUVBL1 and RUVBL2 are druggable MYC effector regulators in neuroblastoma cells
Source: iScience. 2026 Mar 5;29(4):115236. doi: 10.1016/j.isci.2026.115236 (PMC13049659; doi:10.1016/j.isci.2026.115236)
Supplement: Document S1. Figures S1–S13 and Table S1 [file mmc1.pdf]

iScience, Volume *29*

Supplemental information

RUVBL1 and RUVBL2 are druggable MYC effector regulators in neuroblastoma cells

Joachim Tetteh Siaw, Arne Claeys, Wei-Yun Lai, Marcus Borenäs, Elien Hilgert, Sarah-Lee Bekaert, Ellen Sanders, Irem Kaya, Jo Van Dorpe, Frank Speleman, Kaat Durinck, Bengt Hallberg, Ruth H. Palmer, and Jimmy Van den Eynden

Figure S1

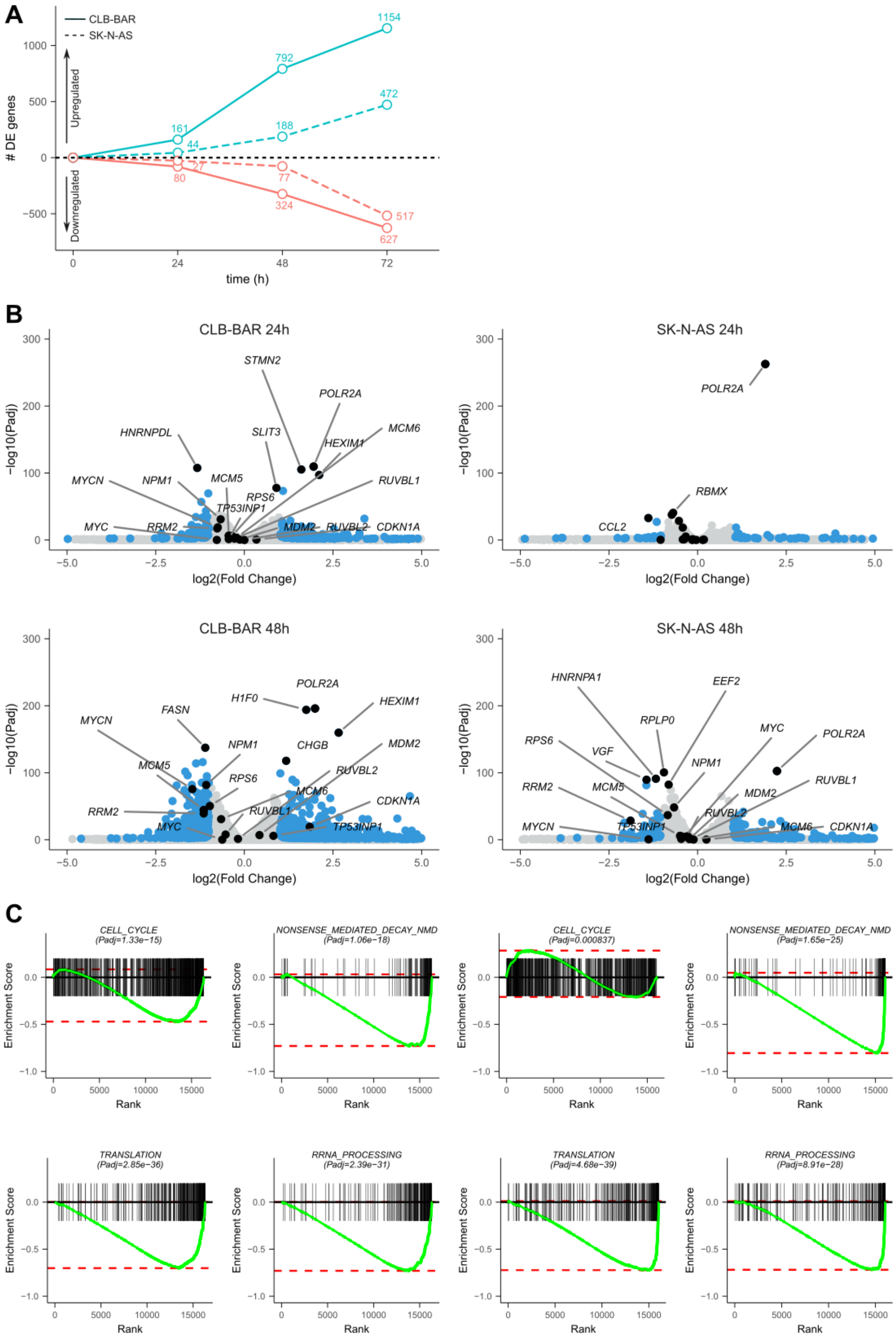

Figure S1. Transcriptomic response to CB-6644 treatment of NB cells, related to Figure 3.

CLB-BAR and SK-N-AS NB cells were treated for 24-72h with CB-6644 250 nM and differential gene expression (DGE) was determined. **A.** Number of up- and downregulated genes (threshold log2FoldChange of ±1 at 1% FDR) for both cell lines and 3 time points as indicated. **B.** Volcano plots showing DGE results for both cell lines after 24h and 48h of treatment as indicated. Differentially expressed genes indicated in blue with top up/downregulated and genes discussed in main text labelled. **C.** GSEA running score plots for 4 Reactome gene sets in both cell lines as indicated. See Table S2 for detailed DGE and GSEA results.

Figure S2

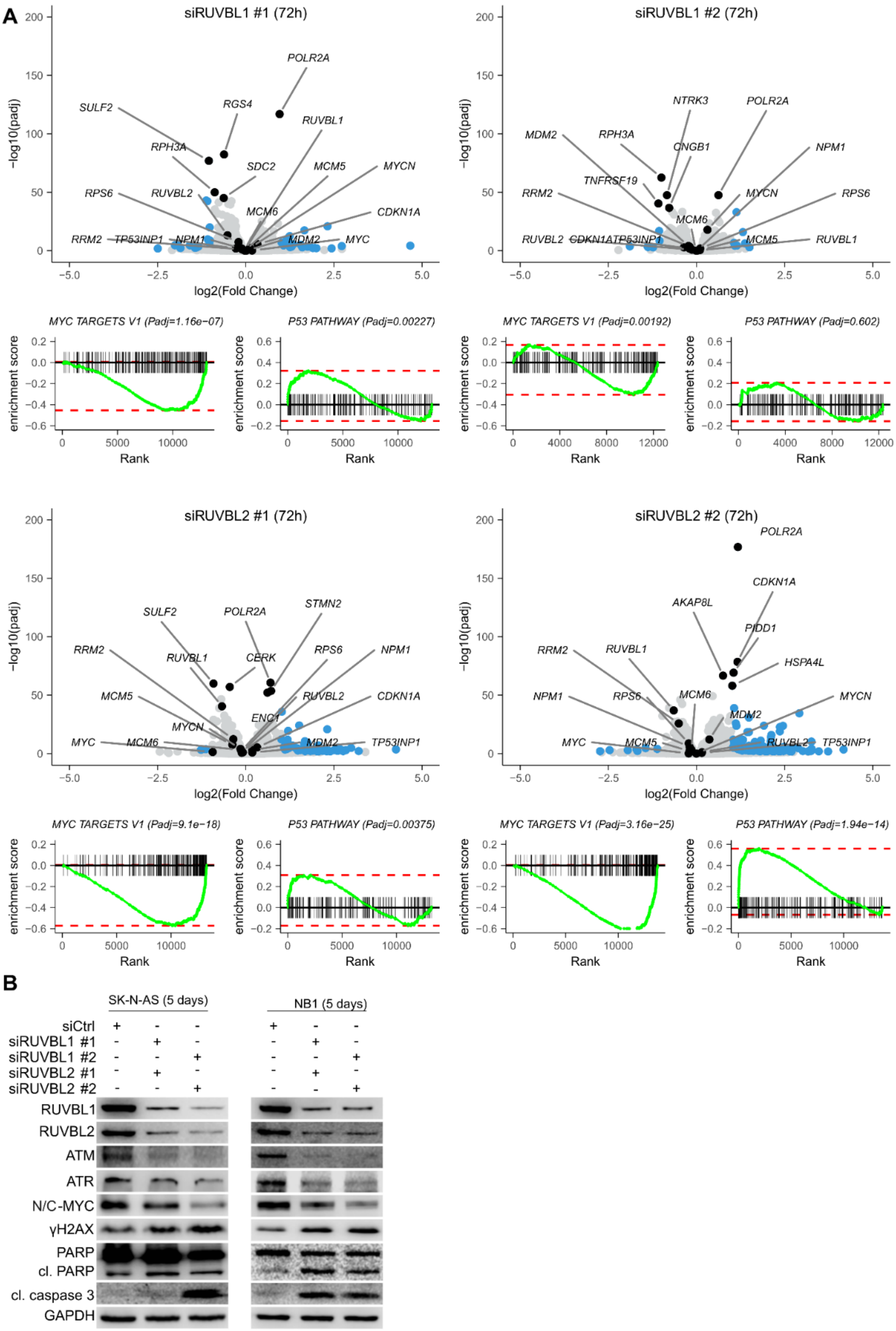

Figure S2. Effect of siRNA-mediated knockdown of *RUVBL1* and *RUVBL2*, related to Figure 3.

A. Volcano plots showing differential gene expression results after treatment of CLB-BAR cells for 72h with 4 siRNAs as indicated. Differentially expressed genes (threshold log2FoldChange of ±1 at 1% FDR) indicated in blue with top up/downregulated and genes discussed in main text labelled. Corresponding GSEA running score plots for 2 Hallmark gene sets shown below each volcano plot. **B.** Western blot showing the effect of siRNA-mediated *RUVBL1* and/or *RUVBL2* knockdown on downstream signaling proteins, 5 days after transfection of two independent NB cell lines as indicated. Blots are representative of two independent experiments. Cl, cleaved. See Fig. S11 for western blot source data.

Figure S3

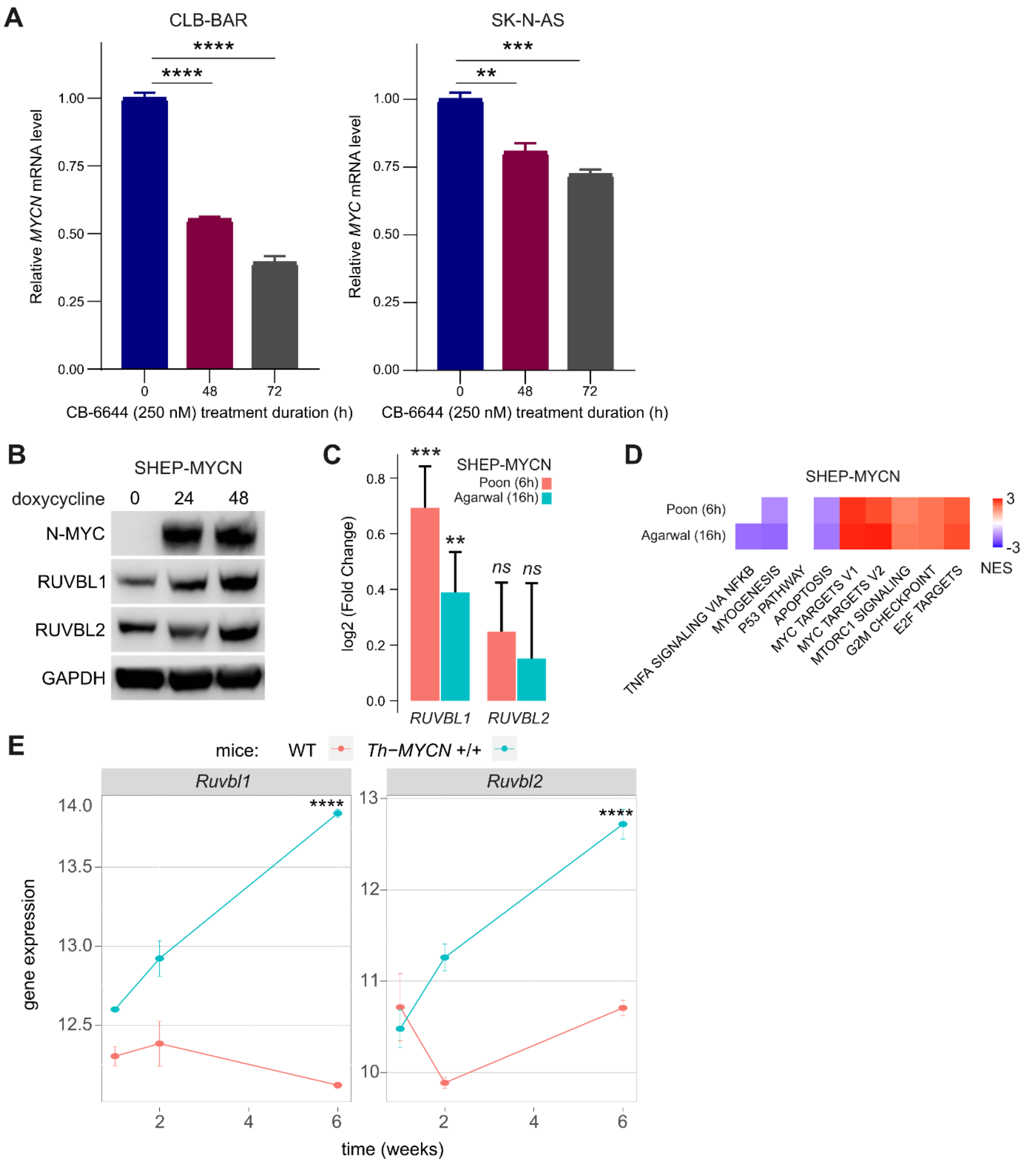

Figure S3. RUVBL1/2 and MYCN correlation in cell lines and MYCN-driven mice models, related to Figure 3.

A. Quantitative PCR was performed after treatment of CLB-BAR and SK-N-AS cells with CB-6644 (250 nM) for 48h and 72h. Bar plots indicating *MYCN* (CLB-BAR) and *MYC* (SK-N-AS) mRNA levels. Results are means +/- SEM of three independent biological replicates. ** *P* value < 0.01, *** *P* value < 0.001, **** *P* value < 0.0001. Unpaired, two-sided Student's t-test. **B.** Western blot showing the effect of doxycycline-induced *MYCN* overexpression on RUVBL1 and RUVBL2 protein expression in SHEP cells. **C-D.** Differential gene expression results of *MYCN* induction in SHEP cells of 2 independent RNA-Seq studies as indicated (time after induction between brackets). **C.** Bar plot showing *RUVBL1* and *RUVBL2* upregulation (log2 (Fold Change) +/- SEM). **, P < 0.01; ***, P < 0.001, ns, non-significant. **D.** Heatmap showing GSEA NES scores for the 2 main clusters as in Fig. 3C. **E.** Time course of *Ruvbl1* and *Ruvbl2* gene expression during control (WT, wild-type) and Th-MYCN-driven tumorigenesis in mice. Data derived from *De Wyn et al.*[1] ****, Padj < 0.0001, moderated t-statistic. See Fig. S12 for western blot source data.

Figure S4

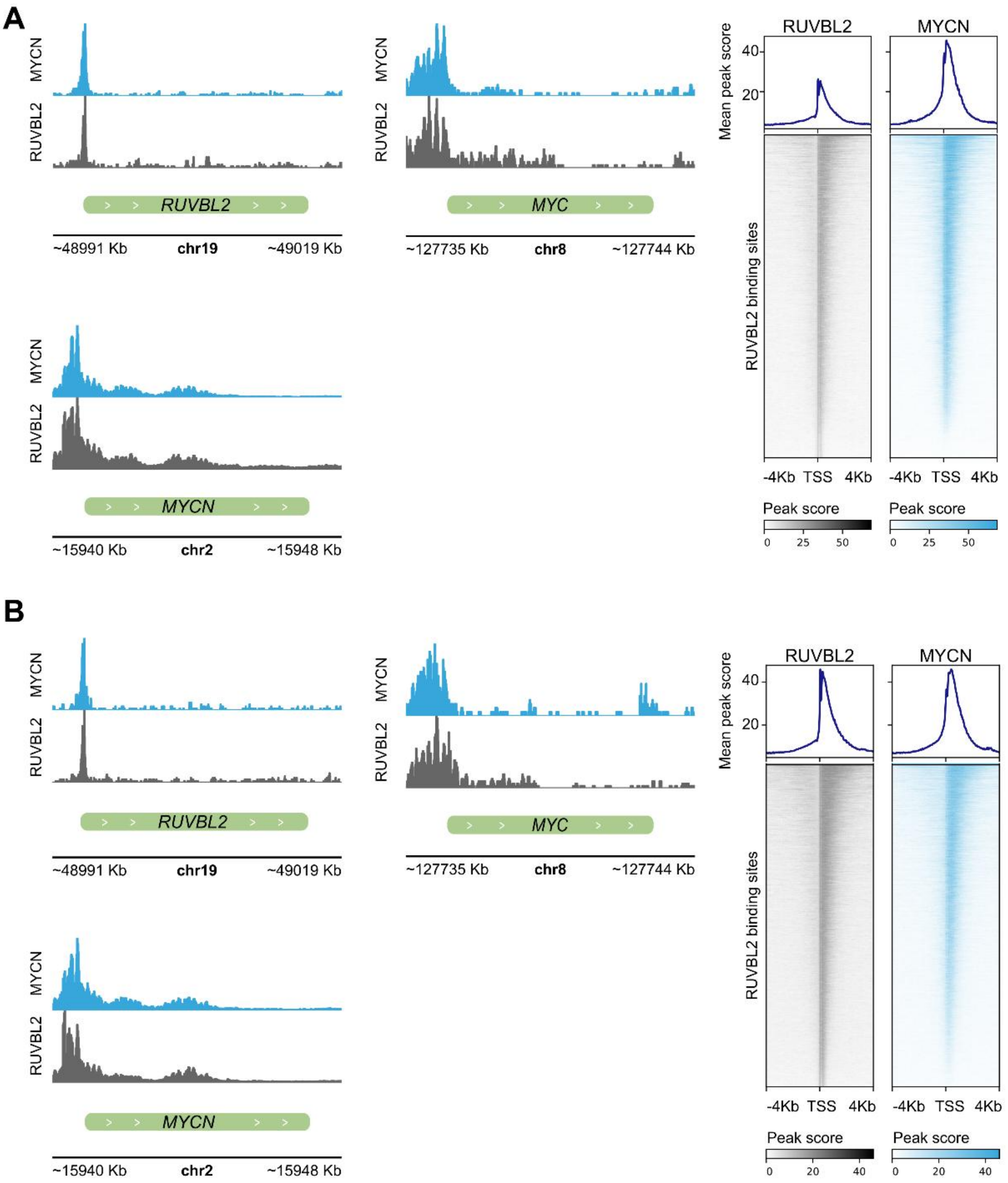

Figure S4. RUVBL2 and MYCN CUT&RUN results in CLB-BAR NB cells, related to Figure 5.
CUT&RUN results as reported in Figure 5 for replicates 2 (**A**) and 3 (**B**).

Figure S5

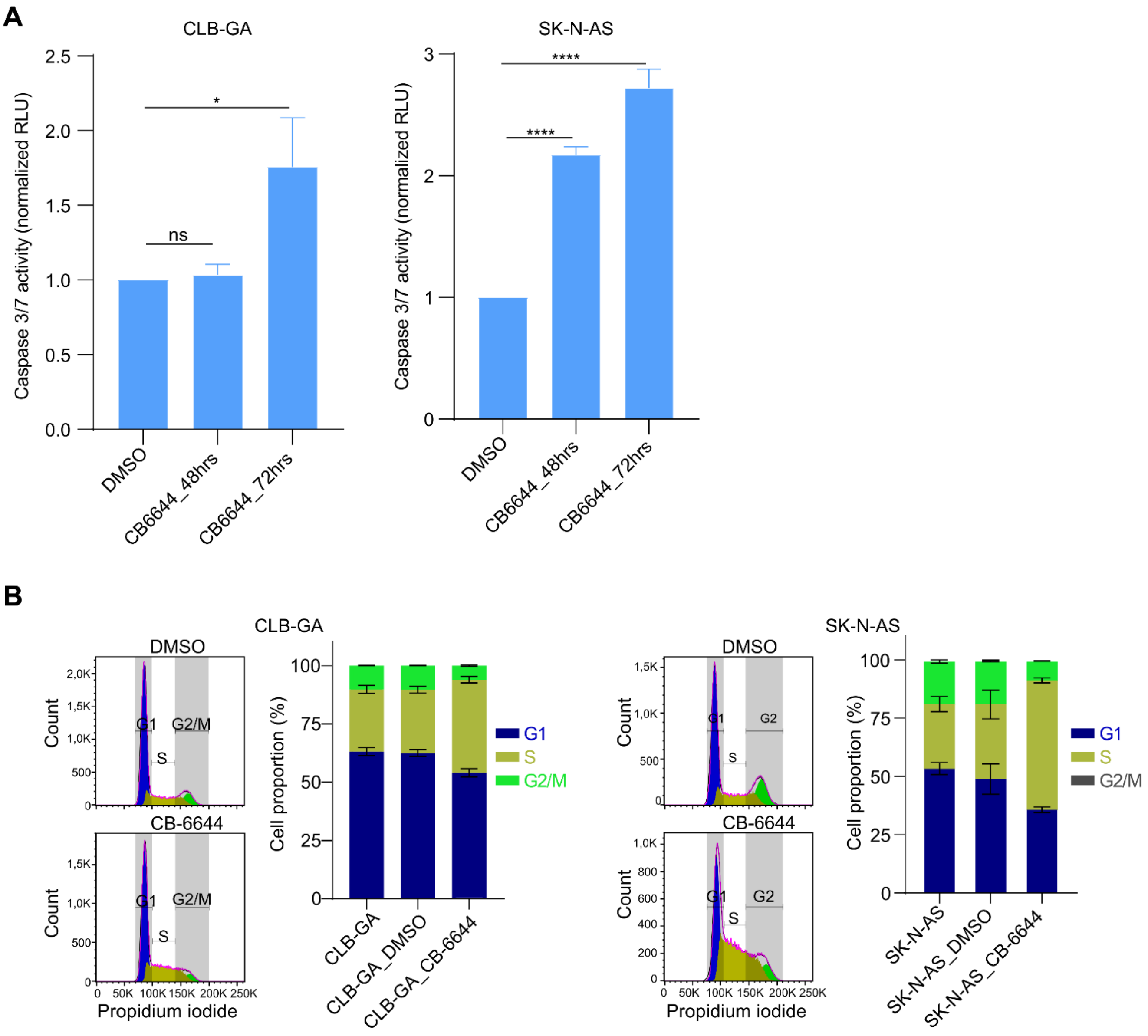

Figure S5. Experimental validation of apoptosis induction and cell cycle alterations upon NB cell treatment with CB-6644, related to Figure 4.

A. CLB-GA and SK-N-AS NB cells were treated with CB-6644 (250 nM) and caspase 3/7 activity was measured after 48h and 72h as indicated in bar plots. Caspase activity was quantified using relative light unit (RLU) and normalized to DMSO (control) groups. Results are means +/- SEM of three independent biological replicates. ns: not significant, * *P* value < 0.05, **** *P* value < 0.0001, unpaired two-sided t-test. **B.** Flow cytometry-based cell cycle analyses of propidium iodide-stained NB cell lines treated for 48h with DMSO or CB-6644 at IC50 concentrations (CLB-GA: 120 nM; SK-N-AS: 250 nM). Stacked bar plots show proportions in each cell cycle phase, at different conditions, for two experimental repeats.

Figure S6

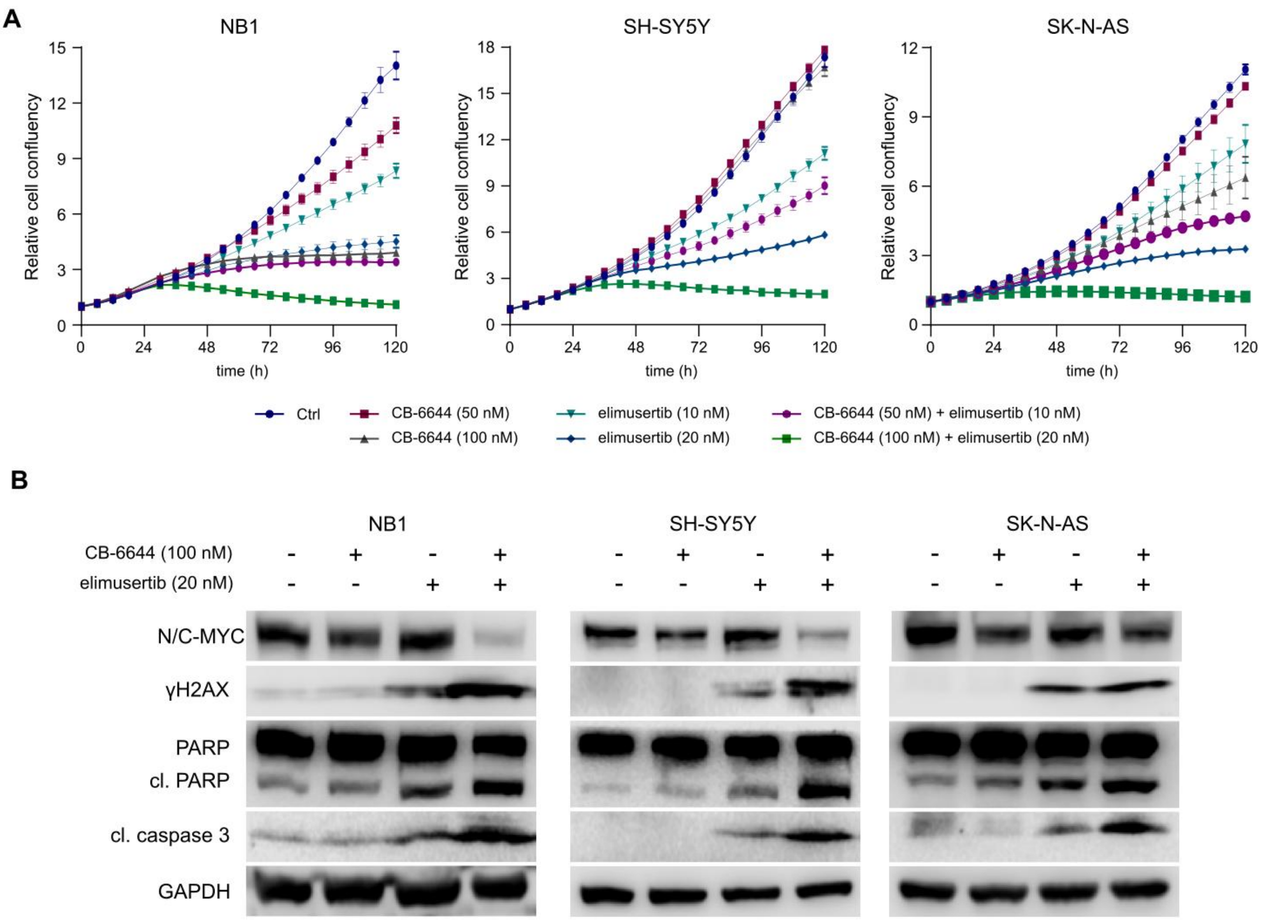

Figure S6. Effect of combined RUVBL1/2 and ATR inhibition on NB cell growth

(A) Time-courses showing the effect of the RUVBL1/2 inhibitor CB-6644 or the ATR inhibitor elimusertib, alone or in combination, on NB cell growth. Cell growth was monitored by scanning cell confluency at regular intervals with IncuCyte® Live Cell Analysis system and normalized to the first scan at time zero. Results are mean +/- SEM of 2 independent biological replicates. **(B)** Western blot showing the effect of CB-6644 +/- elimusertib on MYCN (NB1 and SH-SY5Y), MYC (SK-N-AS), DNA damage (γH2AX) and apoptotic markers (cleaved (cl) caspase 3, PARP) in NB cells. Cells were treated with inhibitors for 48 hrs. See Fig. S13 for western blot source data.

Figure S7

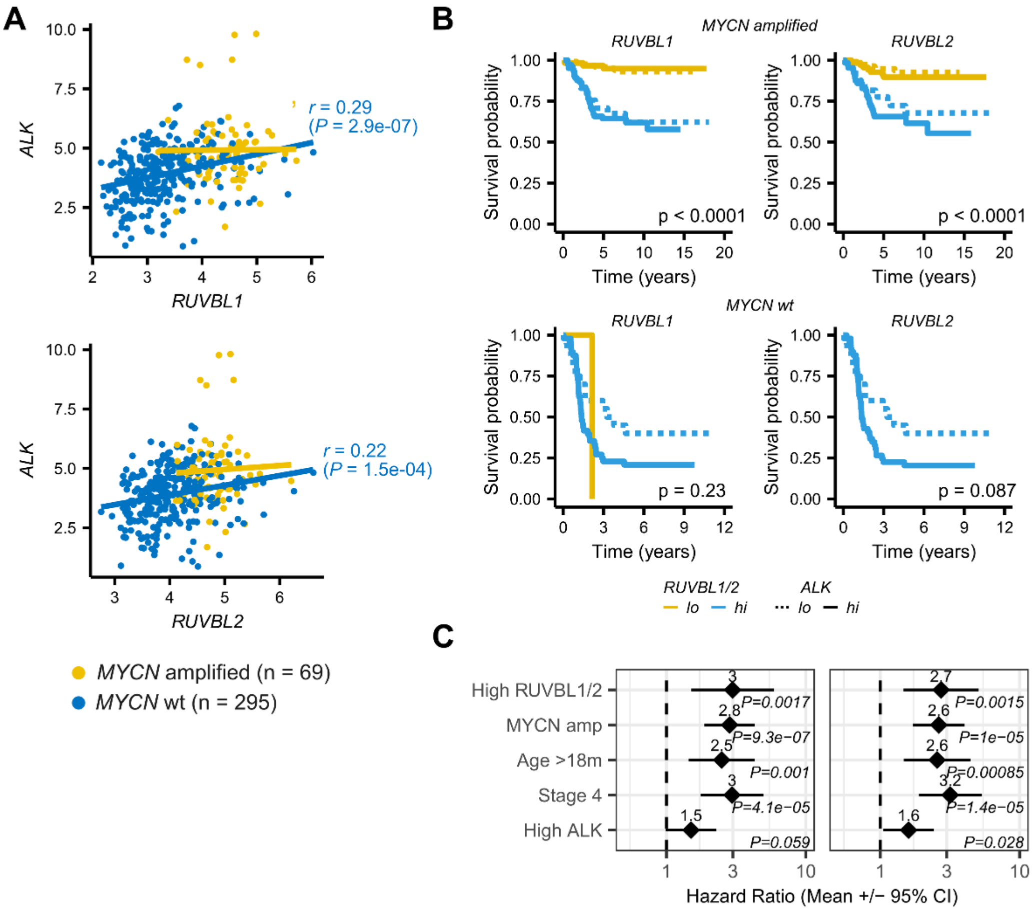

Figure S7. Multivariate survival analysis in primary NB data, related to Figure 6.

A. Correlation plots between *RUVBL1* (top), *RUVBL2* (bottom) and *ALK* gene expression (log2 normalized counts) for *MYCN* amplified and *MYCN* wild-type tumors. Linear regression line and Pearson's correlation coefficient indicated. **B.** Kaplan-Meier survival plots comparing overall survival between patients with high and low *ALK* expression after stratification for *RUVBL1 (left)* and *RUVBL2* (right) expression and for MYCN status, as indicated. High/low *RUVBL1/2* and *ALK* expression defined based on median gene expression. *P* value calculated using log rank test. **C.** Forest plots comparing hazard ratios +/- 95% confidence intervals for 5 variables as indicated. Results were obtained using a Cox proportional hazards multivariate regression analysis.

Figure S8

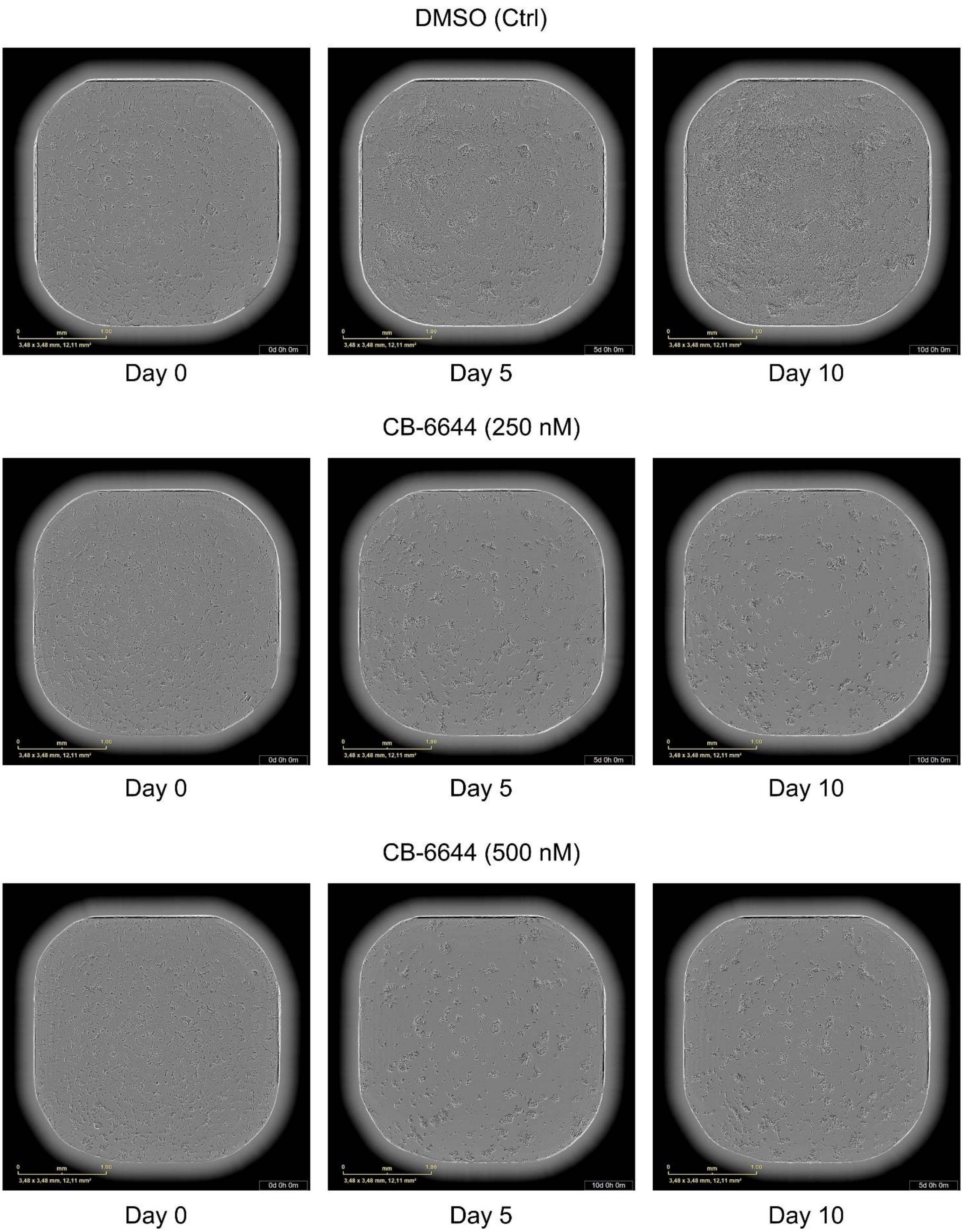

Figure S8. Microscopic images of NB PDX organoids after CB-6644 treatment, related to Figure 6.

Figure S9

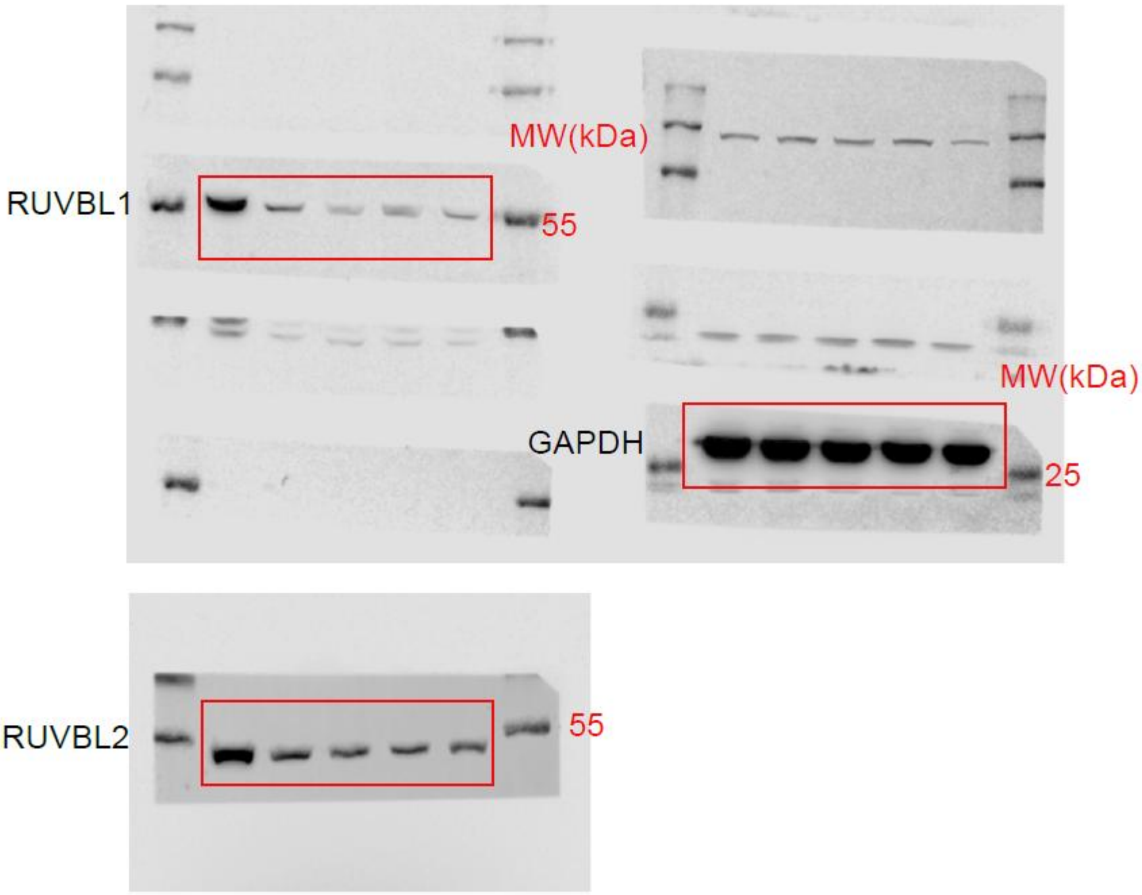

Figure S9. Source data of the western blots shown in Fig. 2B

Western blots shown in Fig. 2B are indicated by red squares. Molecular weights (MW) indicated and labelled on the right.

Figure S10

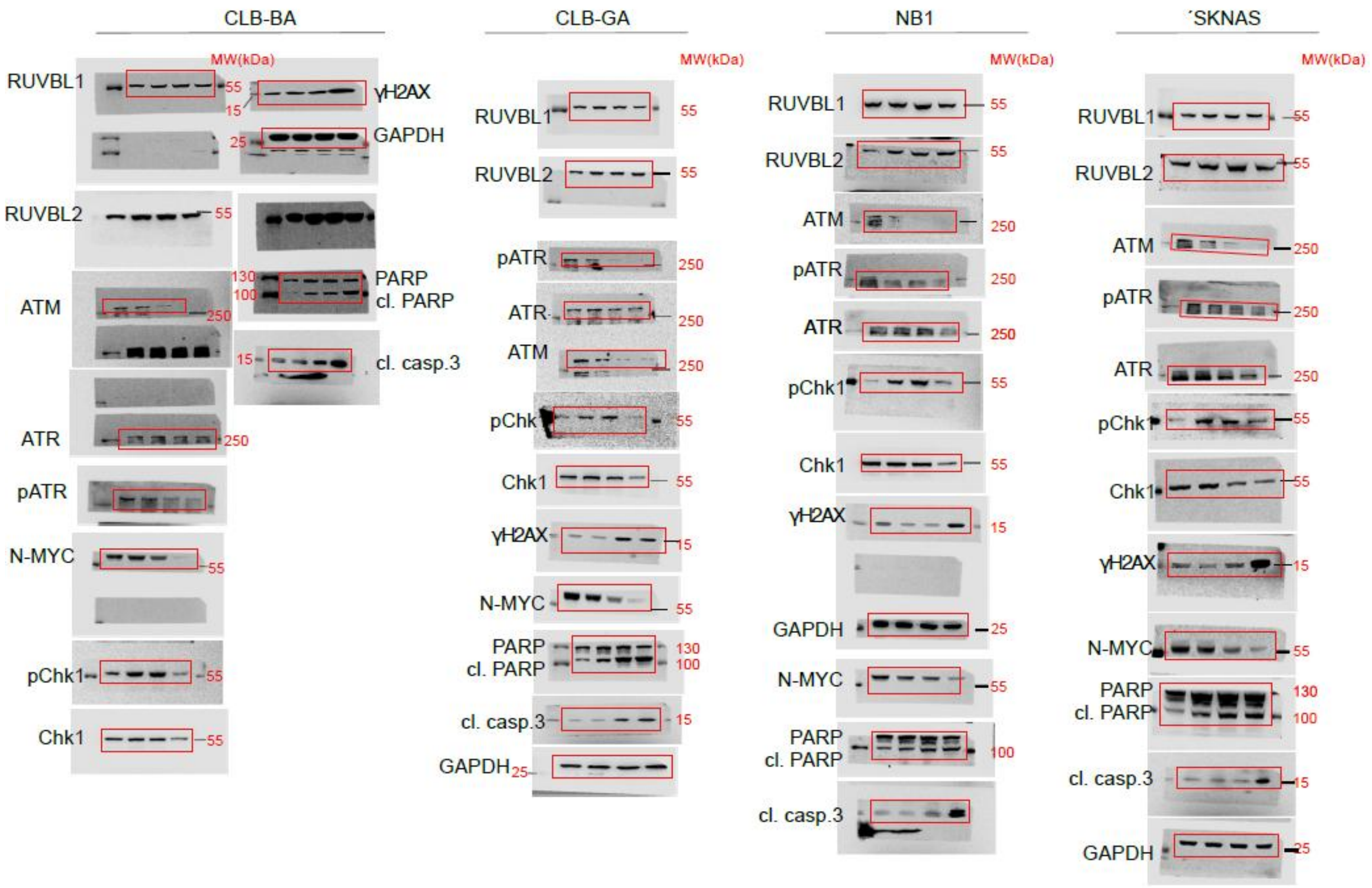

Figure S10. Source data of the western blots shown in Fig. 4

Western blots shown in Fig. 4 are indicated by red squares. Molecular weights (MW) indicated and labelled on the right.

Figure S11

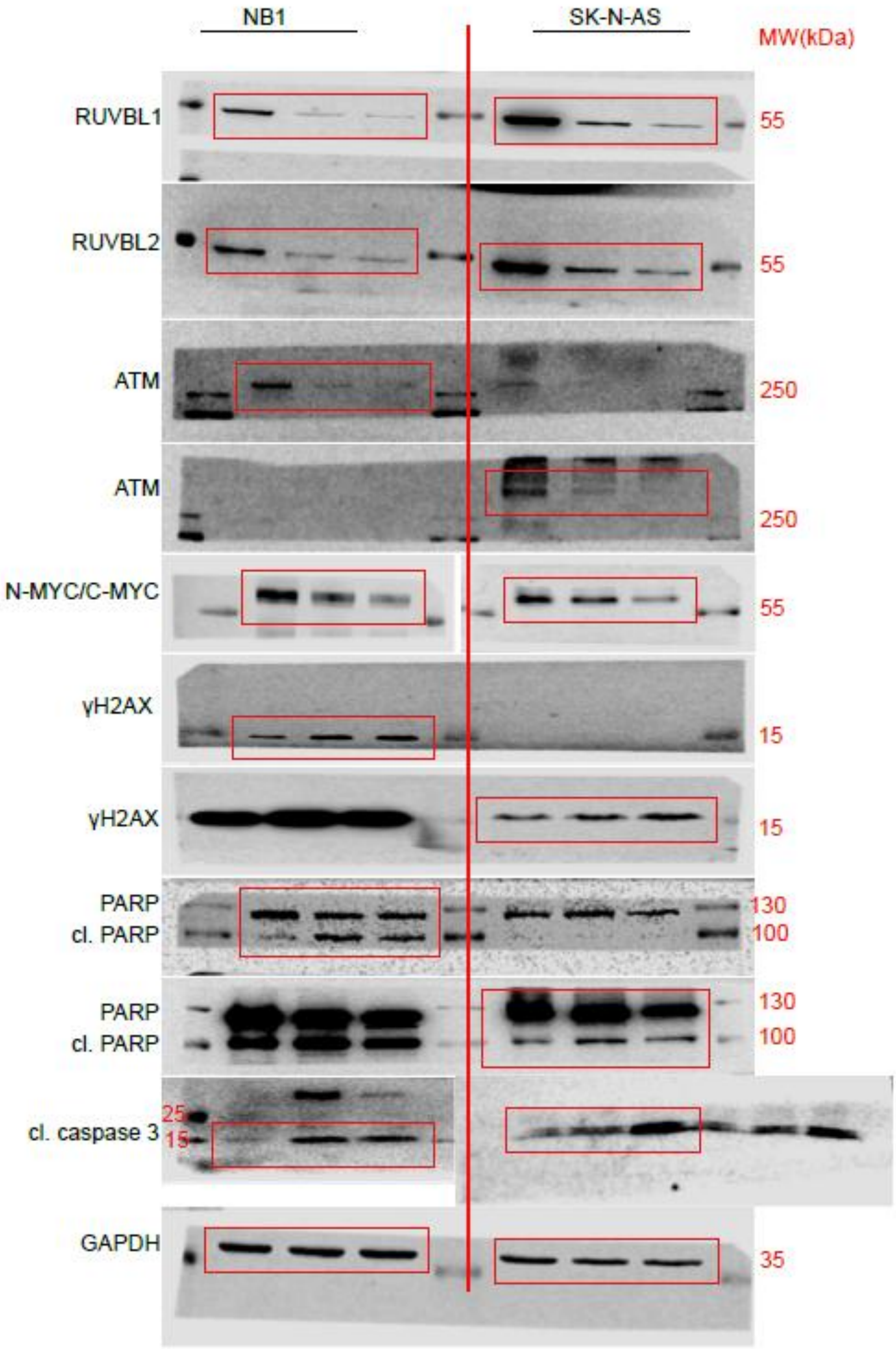

Figure S11. Source data of the western blots shown in Fig. S2B

Western blots shown in Fig. S2B are indicated by red squares. Molecular weights (MW) indicated and labelled on the right.

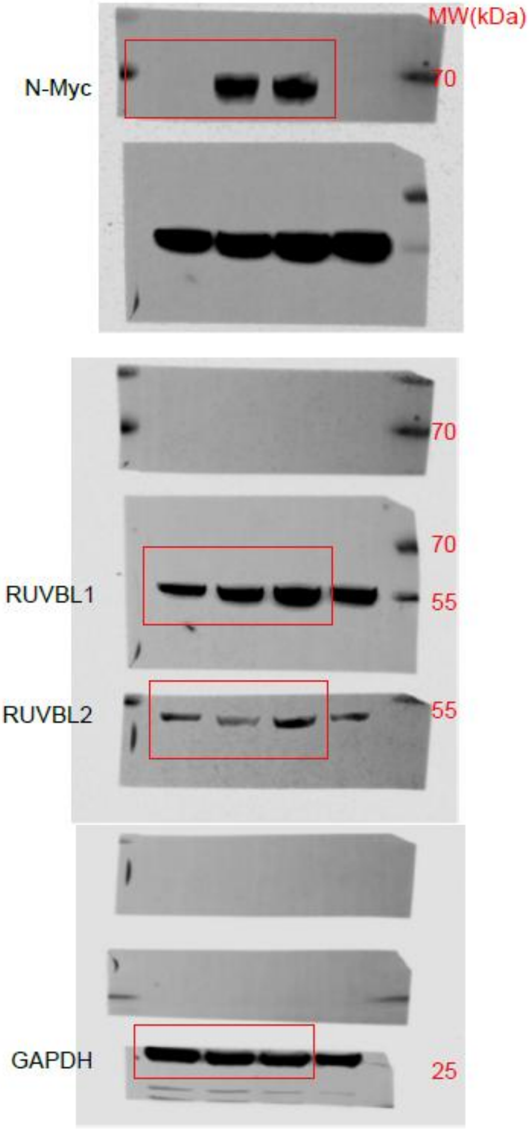

Figure S12. Source data of the western blots shown in Fig. S3B

Western blots shown in Fig. S3B are indicated by red squares. Molecular weights (MW) indicated and labelled on the right.

Figure S13

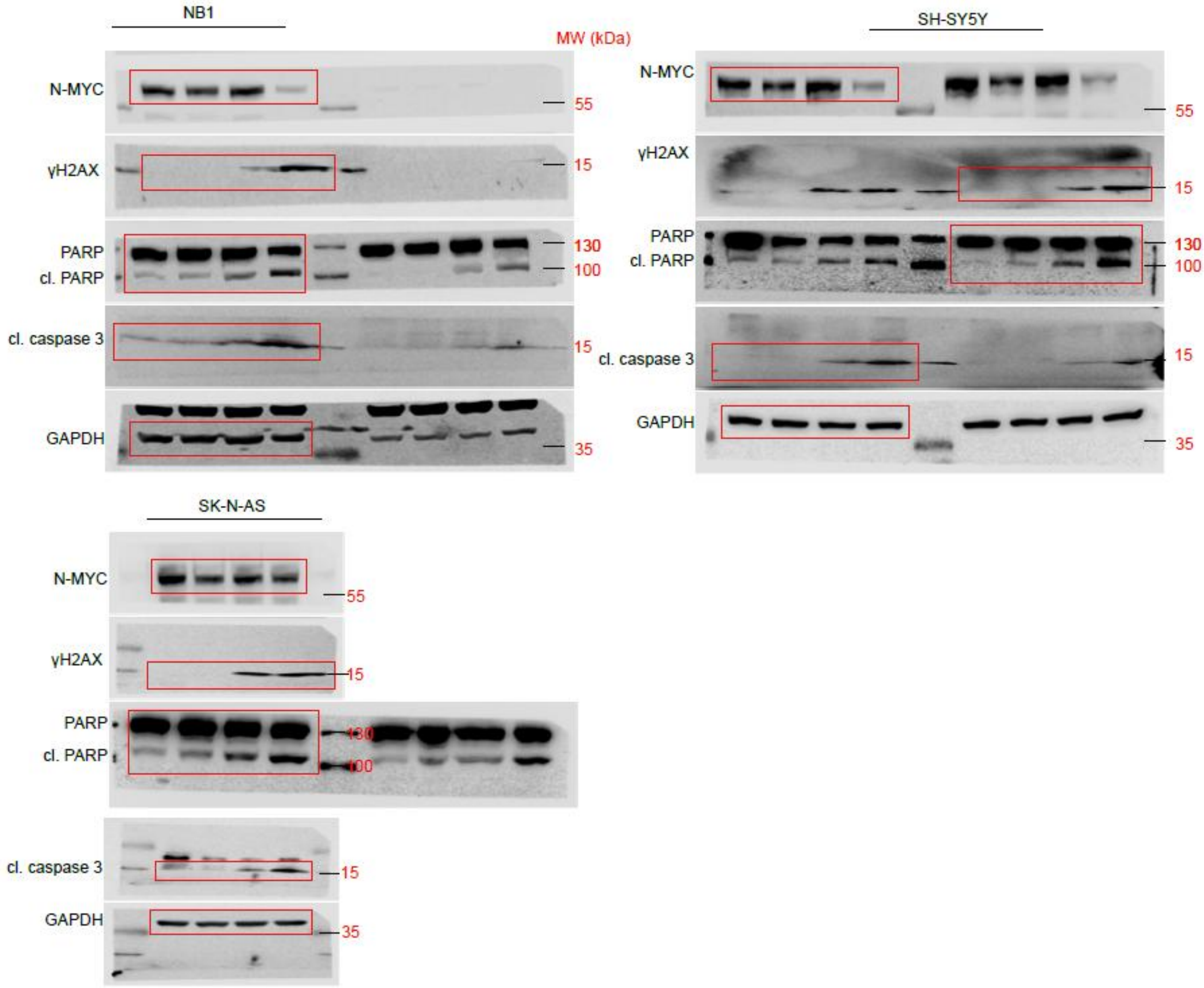

Figure S13. Source data of the western blots shown in Fig. S6B

Western blots shown in Fig. S6B are indicated by red squares. Molecular weights (MW) indicated and labelled on the right.

**Cell line**	***ALK* status**	***MYCN* status**	***TP53* status**	**Other**	**Source**
CLB-BAR	Amplified, deletion of exon 4-11	Amplified	Wild-type		CVCL_9519<br>Umapathy et al.[2]
CLB-GE	Amplified<br>Mutated (F1174V)	Amplified	Wild-type		CVCL_9530<br>Umapathy et al.[2]
CLB-GA	2p-gain with break within *ALK* intron 1<br>Mutated (R1275Q)	Wild-type	Wild-type	11q deletion	CVCL_9529<br>Umapathy et al.[2]<br>De Brouwer et al.[3]
NB1	Amplified	Amplified	Wild-type		CVCL_1440<br>De Brouwer et al.[3]
SH-SY5Y	Mutated (F1174L)	Wild-type	Wild-type		CVCL_0019
SK-N-AS	Wild-type	Wild-type	Deleted	11q deletion, *NRAS* mutated (G61L)	CVCL_1700<br>De Brouwer et al.[3]<br>Goldschneider et al.[4]
SK-N-BE(2)	Wild-type	Amplified	Deleted, Mutated (C135F)	11q deletion	CVCL_0528<br>De Brouwer et al.[3]<br>Goldschneider et al.[4]

Table S1. Genomic profiles of the NB cell lines employed in this study.

Overview of genomic alterations for the NB cell lines used in this study. Cellosaurus accession nr. (www.cellosaurus.org) and other source references indicated.

References

1. De Wyn, J., Zimmerman, M.W., Weichert-Leahey, N., Nunes, C., Cheung, B.B., Abraham, B.J., Beckers, A., Volders, P.-J., Decaesteker, B., Carter, D.R., et al. (2021). MEIS2 is an adrenergic core regulatory transcription factor involved in early initiation of TH-MYCN-driven neuroblastoma formation. Cancers *13*, 4783. https://doi.org/10.3390/cancers13194783.

2. Umapathy, G., Guan, J., Gustafsson, D.E., Javanmardi, N., Cervantes-Madrid, D., Djos, A., Martinsson, T., Palmer, R.H., and Hallberg, B. (2017). MEK inhibitor trametinib does not prevent the growth of anaplastic lymphoma kinase (ALK)–addicted neuroblastomas. Sci. Signaling *10*, eaam7550. https://doi.org/10.1126/scisignal.aam7550.

3. Brouwer, S.D., Preter, K.D., Kumps, C., Zabrocki, P., Porcu, M., Westerhout, E.M., Lakeman, A., Vandesompele, J., Hoebeeck, J., Maerken, T.V., et al. (2010). Meta-analysis of neuroblastomas reveals a skewed ALK mutation spectrum in tumors with MYCN amplification. Clin. Cancer Res. *16*, 4353–4362. https://doi.org/10.1158/1078-0432.CCR-09-2660.

4. Goldschneider, D., Horvilleur, E., Plassa, L.-F., Guillaud-Bataille, M., Million, K., Wittmer-Dupret, E., Danglot, G., de Thé, H., Bénard, J., May, E., et al. (2006). Expression of C-terminal deleted p53 isoforms in neuroblastoma. Nucleic Acids Res. *34*, 5603–5612. https://doi.org/10.1093/nar/gkl619.
